# Associations of percentage energy intake from total, animal and plant protein with overweight/obesity and underweight among adults in Addis Ababa, Ethiopia

**DOI:** 10.1017/S1368980022001100

**Published:** 2022-11

**Authors:** Elena C Hemler, Sabri Bromage, Amare Worku Tadesse, Rachel Zack, Yemane Berhane, Chelsey R Canavan, Wafaie W Fawzi, Walter C Willett

**Affiliations:** 1 Department of Global Health and Population, Harvard T.H. Chan School of Public Health, Boston, MA 02120, USA; 2 Department of Nutrition, Harvard T. H. Chan School of Public Health, Boston, MA, USA; 3 Department of Epidemiology and Biostatistics, Addis Continental Institute of Public Health, Addis Ababa, Ethiopia; 4 Department of Infectious Disease Epidemiology, London School of Hygiene & Tropical Medicine, London, UK; 5 The Greater Boston Food Bank, Boston, MA, USA; 6 Department of Epidemiology, Harvard T.H. Chan School of Public Health, Boston, MA, USA

**Keywords:** Double burden of malnutrition, Animal-source protein, Plant-source protein

## Abstract

**Objective:**

This study investigated associations between types and food sources of protein with overweight/obesity and underweight in Ethiopia.

**Design:**

We conducted a cross-sectional dietary survey using a non-quantitative FFQ. Linear regression models were used to assess associations between percentage energy intake from total, animal and plant protein and BMI. Logistic regression models were used to examine the associations of percentage energy intake from total, animal and plant protein and specific protein food sources with underweight and overweight/obesity.

**Setting:**

Addis Ababa, Ethiopia.

**Participants:**

1624 Ethiopian adults (992 women and 632 men) aged 18–49 years in selected households sampled using multi-stage random sampling from five sub-cities of Addis Ababa.

**Results:**

Of the surveyed adults, 31 % were overweight or obese. The majority of energy intake was from carbohydrate with only 3 % from animal protein. In multivariable-adjusted linear models, BMI was not associated with percentage energy from total, plant or animal protein. Total and animal protein intake were both associated with lower odds of overweight/obesity (OR per 1 % energy increment of total protein 0·92; 95 % CI: 0·86, 0·99; *P* = 0·02; OR per 1 % energy increment of animal protein 0·89; 95 % CI: 0·82, 0·96; *P* = 0·004) when substituted for carbohydrate and adjusted for socio-demographic covariates.

**Conclusion:**

Increasing proportion of energy intake from total protein or animal protein in place of carbohydrate could be a strategy to address overweight and obesity in Addis Ababa; longitudinal studies are needed to further examine this potential association.

Malnutrition is the top risk factor for death and disability in Ethiopia, responsible for 24 % of all deaths^(1)^. The country faces a high double burden of malnutrition, with 22 % of women and 33 % of men underweight, and 8 % of women and 3 % of men overweight or obese. In Ethiopia’s capital city, Addis Ababa, the prevalence of overweight and obesity (29 % of women and 20 % of men) has overtaken that of underweight (13 % of women and 18 % of men)^(2)^. Overweight and obesity, along with unhealthy lifestyles, are important risk factors for non-communicable diseases such as CVD, type 2 diabetes and some cancers^(3)^. Obesity is particularly harmful in low-income settings with high rates of childhood malnutrition, as exposure to undernutrition in early life exacerbates the relationship between obesity and risk of non-communicable diseases^(4)^. As in other urban areas in sub-Saharan Africa (SSA), non-communicable diseases such as diabetes and CVD have become major causes of morbidity and mortality in Addis Ababa, responsible for 31 % of deaths reported by hospitals^(5)^.

Innovative strategies are needed to address the double burden of malnutrition, especially in low-income, urban settings. Increasing the proportion of energy intake from protein could be a potential intervention to simultaneously prevent or treat underweight and overweight. Protein deficiency, contributing to protein-energy malnutrition, is a major cause of disability in Ethiopia^(6)^. In 2013, an estimated 49 % of women and 69 % of men in Ethiopia were below the estimated average requirement for protein intake^(7)^. Protein intake in low-income countries such as Ethiopia tends to be limited, as the majority of energy intake comes from cereal-based staple foods, with on average only 3 % of energy from meat, 11 % from roots and tubers and 6 % from pulses, nuts and oilseeds^(8)^. Low protein intake can lead to stunting and wasting, especially in childhood^(8)^. An analysis of 180 countries found that higher national-level estimates of total protein intake were associated with lower prevalence of child stunting^(9)^. Higher utilisable protein estimates (which take into account the digestibility of the protein sources and the essential amino acid composition) were associated with lower prevalence of child stunting, independent of energy intake^(9)^. One study estimated a potential 1·19-year increase in life expectancy at birth if the adult population of Addis Ababa ate enough protein to meet the required daily amounts and a 0·42–2·0 percentage point decrease in child stunting, with animal-source foods more efficacious than plant-source foods in reducing stunting^(10)^.

While evidence from low-income settings is limited, studies in Western populations suggest that increased protein intake may have favourable effects on body weight and cardiometabolic health. In the USA and Canada, protein intake above the RDA has been associated with lower body weight, more favourable body composition and lower waist circumference^(11,12)^. A meta-analysis of short-term randomised trials suggests that high protein diets (20–35 % of energy from protein) may lower cardiometabolic risk through changes in body composition and/or weight^(13)^. Higher-protein diets often have a relatively low energy density, which can aid appetite suppression, reduce overall food intake, preserve lean body mass and promote weight management^(14)^.

While evidence from short-term studies indicates that overall protein intake may be beneficial for improving cardiometabolic health, it is important to consider potential divergent health effects of distinct protein sources. Traditionally, animal-source protein has been thought to be beneficial for the reduction of undernutrition^(15,16)^, but the relationship between animal protein, plant protein and cardiometabolic health is unclear. In the USA, diets higher in both plant and animal protein, independent of other dietary factors, have been associated with cardiometabolic benefits including decreased BMI and waist circumference^(17)^. In Korea, increased amounts of both plant and animal protein have been found to decrease BMI and waist circumference^(18)^. However, in Belgium, plant protein intake was associated with lower BMI and waist circumference in males and females, while animal protein was associated with higher BMI and higher waist circumference in males^(19)^. Plant protein intake has also been associated with favourable changes in waist circumference in the USA^(20)^ and lower risk of metabolic syndrome in Australia^(21)^. In two large US cohorts, total protein intake was not associated with total mortality, but animal protein was associated with higher mortality and plant protein was associated with lower mortality. However, these findings may reflect in part the types of fat in animal and plant foods and other unhealthy components of animal-source foods commonly consumed in Western settings (such as sodium, nitrates and nitrites found in processed red meat) rather than just the type of protein^(22)^.

Nearly all evidence on protein intake and cardiometabolic health is from high-income countries, which may not be generalisable to low-income settings where animal protein consumption is much lower and food sources of protein differ. While shifts from animal to plant sources of protein are encouraged in Western populations for health and environmental benefits, promotion of animal-source foods in place of carbohydrate in low-resource settings in SSA, where the majority of energy is from starchy carbohydrates, may improve dietary quality, micronutrient intake and have metabolic benefits through reducing glycaemic load^(15,23)^. Ethiopia is currently creating dietary guidelines to address the double burden of malnutrition^(24)^; however, there is a lack of robust dietary data on protein consumption in Ethiopia and a need to clarify how distinct protein sources are related to underweight and overweight in order to form evidence-based guidelines. Therefore, we aimed to examine the associations between proportion of energy from total, animal and plant protein, as well as food sources of protein, with underweight and overweight/obesity in Ethiopian adults.

## Methods

### Household survey

Between January and March 2018, we conducted a cross-sectional study among 1050 urban households in Addis Ababa including 1050 adult women and 635 adult men 18–49 years of age to characterise patterns in protein-source food production, access and consumption. We used multi-stage random sampling to select households from five sub-cities of Addis Ababa, Ethiopia. Eligible households included a woman of reproductive age (18–49) and at least one child between 6 and 59 months of age. If there was an adult male age 18–49 years available at the household, they were also included in the sample. If more than one adult male or adult female was living in the household and present at the time of the interview, one was randomly selected. If the woman of reproductive age was pregnant, she was included in the survey; however, pregnant women were excluded from this analysis (*n* 56). We invited 1083 eligible households to participate and 1050 provided informed consent (response rate of 97 %). The woman of reproductive age completed a household survey including questions on socio-economic status and demographic characteristics. Both the adult man and woman completed a seventy-three-item non-quantitative FFQ assessing the number of days each food was consumed out of the past 7 d, which was administered at the participant’s household by a trained interviewer. Portion sizes and frequencies of intake were not collected. This FFQ was adapted from a semi-quantitative FFQ previously validated for use against two 24 h diet recalls among urban adults in Dar es Salaam, Tanzania^(25)^ and was modified to reflect foods commonly consumed in Ethiopia. Height was measured for all men and women to the nearest 0·1 cm with the subject barefoot using a stadiometer. Weight was measured to the nearest 0·1 kg with the subject barefoot and in light clothes using a SECA digital scale. BMI was calculated as weight in kilograms divided by height in metres squared. Based on BMI, individuals were classified using standard cut-offs as underweight (< 18·5 kg/m^2^), healthy weight (18·5–24·9 kg/m^2^), overweight (25–29·9 kg/m^2)^ or obese (≥ 30 kg/m^2^). All participants had complete height, weight and dietary data.

### Assessment of dietary intake

We calculated energy, protein, carbohydrate and fat intakes using (1) FFQ dietary intake data, (2) nutrient contents from the Ethiopian Food Composition Table^(26)^ and (3) portion sizes for men and women obtained from previous nutrition surveys in Addis Ababa^(27)^. We used the Tanzanian Food Composition Table^(28)^ where Ethiopian estimates were missing. No participants had implausibly high energy intakes (greater than 4500 kcal/18 828 kJ per day), but five participants with energy intakes less than 500 kcal/2092 kJ per day were excluded from the analysis. We estimated the prevalence of inadequate protein intake by applying the estimated average requirements for protein intake for each individual (0·66 g/kg/d for adult men and non-lactating women and 1·05 g/kg/d for lactating women)^(29)^.

### Covariate Measurements

Covariates were collected in the socio-demographic household survey administered to women and included age, highest level of school completed, religion, marital status, employment, household size, indicators of household living standards and household food insecurity. The women’s covariates were used for the men living in their households. All households had complete covariate data. To measure household wealth, participants were categorised into quartiles based on a wealth index that was constructed using principal components analysis and a set of twelve indicators describing household living standards and possessions owned^(30)^. Household food insecurity was assessed using the Household Food Insecurity Access Scale, which is a standardised scale ranging from 0 to 27 based on a nine-item questionnaire including the following food security domains: uncertainty about the household food supply, insufficient quality of food and insufficient food intake. This scale has been used across cultural contexts and categorises households and populations as food secure, mildly food insecure, moderately food insecure or severely food insecure^(31)^.

### Statistical analysis

Descriptive statistics were presented as means and standard deviations; medians and 25th/75th percentiles or proportions. Relationships between socio-demographic characteristics and protein intake were assessed by testing for linear trends in socio-demographic characteristics across quartiles of percentage energy from total protein intake using linear regression (for continuous variables), logistic regression (for binary variables) and ordered multinomial logistic regression (for categorical variables with more than two levels). Linear models adjusting for socio-demographic variables were used to examine associations between percentage energy from total protein, plant protein and animal protein and continuous BMI. Percentage energy from total protein, plant protein and animal protein were examined in linear models as continuous exposures and also in quartiles, with the first quartile representing the lowest percentage energy intake from total, plant or animal protein. We assessed potential nonlinearity in the associations between BMI and percentage energy from total, plant and animal protein by modelling each exposure in multivariate-adjusted models as cubic splines with 2 df.

Logistic regression models were used to examine the associations of percentage energy from total, plant and animal protein (both in quartiles and as continuous exposures) with a binary variable for overweight/obese (defined as BMI ≥ 25 kg/m^2^) and a binary variable for underweight (BMI < 18·5 kg/m^2^) to examine effects of protein sources on underweight and overweight/obesity independently. Overweight/obese and underweight models were run separately excluding underweight participants from models with an outcome of overweight/obesity and vice versa to maintain a comparison group of those with a healthy weight. Analyses were also performed separately by sex because women and men may have different risks of underweight and overweight^(32,33)^. In addition, the top ten food sources of protein intake in the sample were identified and logistic regression models were used to investigate the associations between percentage energy from each protein food with overweight and underweight.

All dietary factors were expressed as nutrient densities (percentage of total energy intake) to examine diet composition without constraining total energy intake, as this may be the primary mediator of how dietary exposures affect outcomes of body weight^(34,35)^. We used these nutrient density models to estimate the effect of substituting percentage energy from total, animal and plant protein for an equal percentage energy from carbohydrate as well as substituting animal for plant protein. In all analyses, multivariable-adjusted models controlled for female’s age, sex, female’s educational attainment (never attended school, completed primary or less, completed higher than primary), if female is married or living with partner (yes, no), wealth index (poorest, poor, wealthy and wealthiest), female’s religion (orthodox Christian or not orthodox Christian), household size (continuous) and female employed (yes, no). Carbohydrate substitution models controlled for the same covariates as the multivariable-adjusted models plus percentage of calories from total fat (continuous). Carbohydrate substitution models with plant protein as the exposure additionally adjusted for percentage energy from animal protein and models with animal protein as the exposure additionally adjusted for percentage energy from plant protein. Animal and plant protein substitution models were also included to estimate the effect of substituting animal for plant protein (or vice versa) by adjusting for all socio-demographic covariates in the multivariable-adjusted models, plus percentage of calories from carbohydrate (continuous) and percentage of calories from fat (continuous).

Secondary analyses examined the associations between percentage energy from total protein, plant protein and animal protein and continuous BMI separately excluding underweight participants and excluding overweight participants as the relationship between protein intake and BMI may differ in the context of overfeeding and underfeeding^(36)^. Secondary analyses were also performed adjusting for total energy intake as a continuous variable in the linear models examining percentage energy from total protein, plant protein and animal protein with continuous BMI; as well as the logistic models with binary outcomes of overweight and underweight. In addition, because 29 % of participants reported currently lactating, secondary analyses were conducted adjusting for lactation status to rule out potential confounding due to postpartum weight retention. To confirm our estimation of substitution effects using the nutrient density models, we also estimated substitutions using the energy partition model^(34)^ by including percentage of energy from fat, carbohydrate and protein as continuous variables in the same multivariable model. For this secondary analysis, the difference in the coefficients for each macronutrient plus their covariance was used to estimate substitution effects^(34)^.

A two-sided probability value < 0·05 was considered to indicate a statistically significant difference. Statistical analyses were conducted using Stata 16·1 (StataCorp LP).

## Results

Characteristics of the survey population are presented in Table 1. After excluding participants with implausible energy intakes (*n* 5) and pregnant women (*n* 56), there were 632 men and 992 women included in the final analysis. Most participants were Orthodox Christian, had completed primary school or higher and were married. According to the Household Food Insecurity Access Scale, 38 % of households were food insecure^(31)^. Female’s age, male sex, Orthodox Christian (*v*. not), married (*v*. not), household size, household wealth quartile, household food security and BMI were all positively associated with percentage energy intake from total protein in bivariate analyses (*P* < 0·05; Table 1). Median energy intake (P25, P75) was 1695 (1353, 1986) kcal/d for men and 1556 (1223, 1890) kcal/d for women. Mean protein intake was 14 % of total energy for men and 14 % of total energy for women. Mean percentage energy from carbohydrates and fat was 69 % and 17 %, respectively. The proportion of participants who did not meet the estimated average requirement for protein intake was 21 % of men, 23 % of non-lactating women and 62 % of lactating women. Plant protein made up 83 % of total protein intake in men and 84 % in women. Major sources of dietary protein for both men and women included teff (36 % of protein), beef meat (12 %), peas (10 %), lentils (9 %) and wheat (5 %). In this sample, 10 % of women and 8 % of men were underweight, 24 % of women and 23 % of men were overweight and 10 % of women and 3 % of men were obese.

**Table 1:** Demographic characteristics of 1624 men and women aged 18–49 in Addis Ababa, Ethiopia by quartiles of percentage energy intake from total protein

	Percentage energy from total protein									
	Q1† (*n* 406)		Q2 (*n* 406)		Q3 (*n* 406)		Q4 (*n* 406)		Overall (*n* 1624)	
	*%*	*n*	*%*	*n*	*%*	*n*	*%*	*n*	*%*	*n*
**Socio-demographic‡**										
Female’s age years**										
Mean	29·1		29·7		29·9		30·6		29·8	
sd	5·6		5·6		5·9		5·7		5·7	
Female**	64·3	261	66·5	270	63·1	256	50·5	205	61·1	992
Female is Orthodox Christian*	2·7	295	73·2	297	77·8	316	78·1	317	75·4	1225
Education										
Never attended school	10·8	44	9·1	37	9·1	37	11·3	46	10·1	164
Completed less than primary	44·8	182	40·4	164	37·4	152	30·5	124	38·3	622
Completed primary or higher	44·3	180	50·5	205	53·4	217	58·1	236	51·6	838
Marital status**										
Married or lives with partner	84·5	343	90·9	369	90·4	367	93·3	379	89·8	1458
Female employed	36·2	147	35·7	145	36·0	146	30·5	124	34·6	562
Household size*										
Mean	4·3		4·5		4·5		4·7		4·5	
sd	1·5		1·6		1·6		1·8		1·6	
Wealth index**										
Poorest	30·0	122	27·6	112	21·7	88	18·2	74	24·4	396
Poor	27·8	113	26·8	109	25·1	102	23·6	96	25·9	420
Wealthy	23·2	94	20·9	85	27·8	113	25·9	105	24·4	397
Wealthiest	19·0	77	24·6	100	25·4	103	32·3	131	25·3	411
Household is food insecure**	40·6	165	44·3	180	37·7	153	27·6	112	37·6	610
**Anthropometric and nutritional**										
BMI*										
Mean	23·1		23·6		23·3		23·9		23·5	
sd	4·0		4·0		3·9		4·3		4·1	
Underweight (BMI < 18·5)	11·6	47	8·4	34	7·1	29	9·4	38	9·1	148
Overweight (25 ≤ BMI < 30)	22·4	91	26·3	107	21·9	89	24·1	98	23·7	385
Obese (BMI ≥ 30)	5·7	23	7·9	32	5·4	22	9·6	39	7·1	116
Total energy intake (kcal/d)										
Median	1287		1621		1706		1782		1613	
P25, P75	1027, 1616		1328, 1911		1397, 2068		1438, 2135		1279, 1939	
% Energy from plant protein										
Mean	11·1		11·8		12·1		11·9		11·7	
sd	1·1		1·6		1·9		2·3		1·8	
% energy from animal protein										
Mean	0·8		1·9		2·6		4·6		2·5	
sd	1·1		1·6		1·9		2·7		2·4	
Protein intake (g/d)										
Median	38·3		54·9		62·9		73·4		57·0	
P25, P75	29·7, 49·5		44·4, 65·6		52·0, 76·3		60·5, 87·0		43·2, 72·6	
**Estimated average requirements by population group**										
% of men below the estimated average requirement (EAR) for protein intake§ (*n* 632 men)	60·0	87	16·2	22	7·3	11	5·0	10	20·6	130
% of non-lactating women below the EAR for protein intake§ (*n* 529 non-lactating women)	55·5	76	20·1	28	10·5	14	4·2	5	23·3	123
% of lactating women below the EAR for protein intake|| (*n* 463 lactating women)	88·7	110	66·4	87	43·9	54	41·2	35	61·8	286

* *P* < 0·05.

** *P* < 0·001.

† Demographic characteristics are presented by quartiles of percent energy from protein intake, with the first quartile representing the lowest percent energy intake from protein.

‡ Data are presented as mean ± sd or median (P25, P75) for continuous measures and % (n) for categorical measures.

§ EAR for protein intake are 0·66 g/kg/d for adult men and women aged 19–70 years.

|| EAR for protein intake are 1.05 g/kg/d for lactating women.

In linear models adjusted for socio-demographic covariates, percentage energy from total protein was not associated with continuous BMI (Table 2). A greater percentage energy from plant protein was associated with lower BMI in linear models adjusted for age and sex, but this was NS after controlling for covariates. In age- and sex-adjusted linear models, a greater percentage energy from animal protein was positively and significantly associated with increased BMI (*β* for 1 % energy increment 0·15; 95 % CI: 0·07, 0·23; *P* < 0·001). However, this was NS after adjusting for socio-demographic characteristics. Of all covariates, wealth and education were strong confounders of this relationship and inclusion of either in the model nullified the positive association between animal protein and BMI. Additionally adjusting for percentage energy from fat (which changes the model into a substitution of animal protein for carbohydrate) flipped the association to inverse, although this was NS (*β* for 1 % energy increment –0·09; 95 % CI: –0·22, 0·05; *P* = 0·20) (Table 2). There was no evidence of nonlinearity for the associations between BMI and percentage energy from total protein, plant protein and animal protein in multivariate-adjusted models (see online supplementary material, Supplemental Fig. 1) or multivariate-adjusted carbohydrate substitution models (see online supplementary material, Supplemental Fig. 2).

**Table 2:** Beta coefficients (95 % CI) for percentage energy intake from total protein, plant and animal protein in relation to BMI among 1624 adults in Addis Ababa, Ethiopia

		Q2		Q3		Q4		Per 1 % difference‡		
	Q1†	Beta coefficients	95 % CI	Beta coefficients	95 % CI	Beta coefficients	95 % CI	Beta coefficients	95 % CI	*P*_trend_
Percentage energy from total protein										
Mean % energy	11·8	13·6		14·8		16·5		14·2		
Age- and sex-adjusted§	0·0 (Ref)	0·38	–0·16, 0·92	0·05	–0·49, 0·59	0·61*	0·06, 1·15	0·09	–0·02, 0·19	0·07
Multivariable-adjusted||	0·0 (Ref)	0·22	–0·31, 0·75	−0·18	–0·71, 0·35	0·24	–0·30, 0·78	0·01	–0·10, 0·11	0·65
Multivariable-adjusted carbohydrate substitution¶	0·0 (Ref)	0·11	–0·43, 0·65	−0·33	–0·88, 0·22	−0·03	–0·62, 0·56	−0·06	–0·18, 0·06	0·60
Percentage energy from plant protein										
Mean % energy	9·4	11·1		12·3		14·1		11·7		
Age- and sex–adjusted	0·0 (Ref)	−0·47	–1·01, 0·07	−0·42	–0·96, 0·12	−0·71*	–1·25, –0·17	−0·17*	–0·27, –0·07	0·01
Multivariable-adjusted	0·0 (Ref)	−0·27	–0·80, 0·26	−0·04	–0·57, 0·50	−0·04	–0·60, 0·51	−0·02	–0·13, 0·08	0·94
Multivariable-adjusted carbohydrate substitution	0·0 (Ref)	−0·21	–0·78, 0·36	−0·05	–0·65, 0·55	−0·04	–0·70, 0·62	−0·03	–0·17, 0·10	0·93
Multivariable-adjusted animal protein substitution††	0·0 (Ref)	−0·12	–0·67, 0·43	0·09	–0·46, 0·64	0·13	–0·47, 0·73	0·01	–0·11, 0·13	0·54
Percentage energy from animal protein										
Mean % energy	0·0	1·2		2·9		5·7		2·5		
Age- and sex-adjusted	0·0 (Ref)	0·82*	0·28, 1·36	0·66*	0·13, 1·20	1·00**	0·46, 1·54	0·15**	0·07, 0·23	< 0·01
Multivariable-adjusted	0·0 (Ref)	0·47	–0·07, 1·01	0·09	–0·46, 0·65	0·12	–0·45, 0·70	0·02	–0·07, 0·11	0·89
Multivariable-adjusted carbohydrate substitution	0·0 (Ref)	0·27	–0·28, 0·83	−0·30	–0·93, 0·32	−0·56	–1·33, 0·21	−0·09	–0·22, 0·05	0·07
Multivariable-adjusted plant protein substitution‡‡	0·0 (Ref)	0·27	–0·29, 0·83	−0·28	–0·89, 0·33	−0·51	–1·23, 0·21	−0·08	–0·20, 0·03	0·06

* *P* < 0·05.

** *P* < 0·001.

† Percentage energy from total protein, plant protein and animal protein were examined in linear models as continuous exposures and also in quartiles; Q1 represents the lowest percent energy intake from total, plant or animal protein.

‡ Beta coefficients are in kg/m^2^ per 1 % difference in percent energy

§ The age- and sex-adjusted model is adjusted for female’s age (years, continuous) and sex (female, male)

|| The multivariable-adjusted model is adjusted for female’s age, sex, female’s educational attainment (never attended school, completed primary or less, completed higher than primary), if female is married or living with partner (yes, no), wealth index (poorest, poor, wealthy, wealthiest), female’s religion (orthodox Christian or not orthodox Christian), household size (continuous) and female employed (yes, no).

¶ The multivariable-adjusted carbohydrate substitution model is the multivariable-adjusted model additionally adjusted for percentage of calories from total fat (continuous). When plant protein is the exposure, the model is additionally adjusted for percentage of calories from animal protein. When animal protein is the exposure, the model is additionally adjusted for percentage of calories from plant protein.

†† The multivariable-adjusted animal protein substitution model is adjusted for female’s age, sex, percentage of calories from fat, percentage of calories from carbohydrate (continuous), female’s educational attainment, if female is married or living with partner, wealth index, female’s religion, household size and female employed.

‡‡ The multivariable-adjusted plant protein substitution model is adjusted for the same covariates as the multivariable-adjusted animal protein substitution model.

Total protein intake was associated with lower odds of overweight/obesity (OR per 1 % energy increment 0·92; 95 % CI: 0·86, 0·99; *P* = 0·02) compared with healthy weight, after adjusting for age, sex, percentage energy from total fat, educational attainment, marital status, wealth index, religion, household size and employment status (Table 3). Participants in the third quartile of percentage energy from total protein had a lower odds of overweight/obesity compared with those in the lowest quartile (OR 0·69; 95 % CI: 0·49, 0·97; *P* = 0·04) when substituting protein for carbohydrate, but no significant associations were observed when comparing the highest and lowest quartiles (OR 0·89; 95 % CI: 0·62, 1·28; *P* = 0·54). These results were consistent after adjustment for lactation status. In fully adjusted models, total protein was associated with a lower odds of overweight/obesity when substituting for carbohydrate (OR per 1 % energy increment 0·88; 95 % CI: 0·81, 0·97; *P* = 0·009) in women, but no significant associations were observed in men (OR per 1 % energy increment 0·99; 95 % CI: 0·88, 1·12; *P* = 0·91) (see online supplementary material, Supplemental Table 1). In multivariable-adjusted carbohydrate substitution models, total protein intake was not significantly associated with odds of underweight (compared with healthy weight) in the overall sample (OR per 1 % energy increment 0·95; 95 % CI: 0·85, 1·07; *P* = 0·40), in women (OR per 1 % energy increment 0·92; 95 % CI: 0·80, 1·06; *P* = 0·24) or in men (OR per 1 % energy increment 1·09; 95 % CI: 0·89, 1·32; *P* = 0·39) (Table 3; see online supplementary material, Supplemental Table 1).

**Table 3:** OR (95 % CI) for percentage energy intake from total protein, plant protein and animal protein in relation to prevalence of underweight or overweight/obesity among 1624 adults in Addis Ababa, Ethiopia

		Q2		Q3		Q4		Per 1 % difference		
	Q1†	OR	95 % CI	OR	95 % CI	OR	95 % CI	OR	95 % CI	*P*_trend_
Percentage energy from total protein										
Overweight/Obese‡, *n* 1476, 501 overweight or obese										
Age- and sex-adjusted§	1·00 (Ref)	1·24	0·91, 1·70	0·86	0·63, 1·19	1·25	0·92, 1·74	1·00	0·94, 1·07	0·46
Multivariable-adjusted||	1·00 (Ref)	1·13	0·82, 1·56	0·75	0·54, 1·04	1·02	0·73, 1·41	0·96	0·90, 1·02	0·58
Multivariable-adjusted										
Carbohydrate substitution¶	1·00 (Ref)	1·07	0·77, 1·48	0·69*	0·49, 0·97	0·89	0·62, 1·28	0·92*	0·86, 0·99	0·21
Underweight‡, *n* 1123, 148 underweight										
Age- and sex-adjusted	1·00 (Ref)	0·79	0·49, 1·29	0·59*	0·36, 0·98	0·98	0·61, 1·57	0·96	0·87, 1·06	0·56
Multivariable-adjusted	1·00 (Ref)	0·85	0·52, 1·38	0·63	0·38, 1·05	1·03	0·63, 1·68	0·97	0·88, 1·07	0·71
Multivariable-adjusted										
Carbohydrate substitution	1·00 (Ref)	0·83	0·51, 1·36	0·62	0·37, 1·04	0·98	0·57, 1·67	0·95	0·85, 1·07	0·56
Percentage energy from plant protein										
Overweight/obese, *n* 1476, 501 overweight or obese										
Age- and sex-adjusted	1·00 (Ref)	0·76	0·56, 1·04	0·88	0·64, 1·19	0·78	0·57, 1·07	0·94	0·89, 1·00	0·20
Multivariable-adjusted	1·00 (Ref)	0·82	0·60, 1·13	1·05	0·76, 1·45	1·10	0·78, 1·53	1·01	0·95, 1·08	0·40
Multivariable-adjusted										
Carbohydrate substitution	1·00 (Ref)	0·76	0·54, 1·07	0·90	0·63, 1·29	0·90	0·60, 1·34	0·96	0·88, 1·05	0·85
Multivariable-adjusted										
Animal protein substitution**	1·00 (Ref)	0·89	0·64, 1·23	1·13	0·81, 1·57	1·27	0·88, 1·82	1·05	0·97, 1·13	0·11
Underweight *n* 1123, 148 underweight										
Age- and sex-adjusted	1·00 (Ref)	1·00	0·59, 1·70	1·50	0·91, 2·46	1·29	0·77, 2·14	1·08	0·98, 1·19	0·19
Multivariable-adjusted	1·00 (Ref)	0·97	0·56, 1·66	1·37	0·83, 2·28	1·08	0·64, 1·84	1·04	0·94, 1·15	0·58
Multivariable-adjusted										
Carbohydrate substitution	1·00 (Ref)	0·89	0·50, 1·59	1·18	0·67, 2·09	0·89	0·48, 1·68	1·00	0·88, 1·13	0·87
Multivariable-adjusted										
Animal protein substitution	1·00 (Ref)	1·03	0·59, 1·81	1·45	0·86, 2·45	1·24	0·69, 2·21	1·08	0·96, 1·21	0·31
Percentage energy from animal protein										
Overweight/obese *n* 1476, 501 overweight or obese										
Age- and sex-adjusted	1·00 (Ref)	1·40*	1·02, 1·92	1·31	0·95, 1·81	1·29	0·93, 1·77	1·04	0·99, 1·08	0·26
Multivariable-adjusted	1·00 (Ref)	1·13	0·81, 1·58	0·92	0·66, 1·30	0·79	0·55, 1·12	0·96	0·92, 1·02	0·07
Multivariable-adjusted										
Carbohydrate substitution	1·00 (Ref)	0·99	0·70, 1·40	0·71	0·49, 1·05	0·50*	0·31, 0·81	0·89*	0·82, 0·96	< 0·01
Multivariable-adjusted										
Plant protein substitution††	1·00 (Ref)	1·01	0·71, 1·42	0·74	0·51, 1·09	0·55*	0·35, 0·86	0·91*	0·85, 0·98	< 0·01
Underweight *n* 1123, 148 underweight										
Age- and sex-adjusted	1·00 (Ref)	0·91	0·56, 1·45	0·85	0·53, 1·38	0·70	0·42, 1·15	0·92	0·85, 1·00	0·16
Multivariable-adjusted	1·00 (Ref)	0·96	0·59, 1·56	0·96	0·58, 1·59	0·85	0·49, 1·47	0·95	0·87, 1·04	0·59
Multivariable-adjusted										
Carbohydrate substitution	1·00 (Ref)	0·91	0·55, 1·51	0·90	0·50, 1·59	0·78	0·37, 1·65	0·90	0·79, 1·03	0·54
Multivariable-adjusted										
Plant protein substitution	1·00 (Ref)	0·92	0·56, 1·52	0·89	0·51, 1·56	0·77	0·39, 1·53	0·91	0·81, 1·03	0·47

* *P* < 0·05

† Percentage energy intake from total protein, plant protein and animal protein were examined in logistic regression models as continuous exposures and also in quartiles; Q1 represents the lowest percentage energy intake from total, plant or animal protein.

‡ Overweight/obese is defined as BMI ≥ 25 and is compared with a reference category of non-overweight/obese (BMI < 25). All underweight participants (*n* 148) were excluded from the overweight analysis. Underweight is defined as BMI < 18·5 and is compared with a reference category of non-underweight (BMI ≥ 18·5). All overweight participants (*n* 501) were excluded from the underweight analysis.

§ The age- and sex-adjusted model is adjusted for female’s age (years, continuous) and sex (female, male)

|| The multivariable-adjusted model is adjusted for female’s age, sex, female’s educational attainment (never attended school, completed primary or less, completed higher than primary), if female is married or living with partner (yes, no), wealth index (poorest, poor, wealthy, wealthiest), female’s religion (orthodox Christian or not orthodox Christian), household size (continuous) and female employed (yes, no).

¶ The multivariable-adjusted carbohydrate substitution model is the multivariable-adjusted model additionally adjusted for percentage of calories from total fat (continuous). When plant protein is the exposure, the model is additionally adjusted for percentage of calories from animal protein. When animal protein is the exposure, the model is additionally adjusted for percentage of calories from plant protein.

** The multivariable-adjusted animal protein substitution model is adjusted for female’s age, sex, percentage of calories from fat, percentage of calories from carbohydrate (continuous), female’s educational attainment, if female is married or living with partner, wealth index, female’s religion, household size and female employed.

†† The multivariable-adjusted plant protein substitution model is adjusted for the same covariates as the multivariable-adjusted animal protein substitution model.

Among men and women combined, plant protein intake was not significantly associated with odds of underweight or overweight/obesity compared with healthy weight (Table 3). However, plant protein intake was associated with increased odds of overweight/obesity in women when substituted for animal protein (OR per 1 % energy increment 1·11; 95 % CI: 1·01, 1·22; *P* = 0·03), but not when substituted for carbohydrate (OR per 1 % energy increment 0·96; 95 % CI: 0·86, 1·07; *P* = 0·50) (see online supplementary material, Supplemental Table 1). Greater animal protein intake was significantly associated with lower odds of overweight in fully adjusted models when substituted for total carbohydrate (OR per 1 % energy increment 0·89; 95 % CI: 0·82, 0·96; *P* = 0·004) and for plant protein (OR per 1 % energy increment 0·91; 95 % CI: 0·85, 0·98; *P* = 0·008) (Table 3). Participants in the highest quartile of animal protein intake had a 50 % (95 % CI: 19, 69; *P* = 0·005) lower odds of overweight/obesity compared with those in the lowest quartile, substituting animal protein for carbohydrate. These results were consistent after adjustment for lactation status. Women had 68 % (95 % CI: 41, 83; *P* < 0·001) lower odds of overweight/obesity comparing the highest to lowest quartile of animal protein intake, substituted for carbohydrate, but this association was not seen in men. No associations between animal protein intake and underweight were observed in the fully adjusted models overall, in women, or in men (Table 3; see online supplementary material, Supplemental Table 1).

In food-based analyses, percentage energy from most commonly consumed protein sources was not related to underweight or overweight/obesity (Table 4). In fully adjusted models, a greater percentage energy from milk was associated with lower odds of underweight but the CI was wide (OR per 5 % energy increment 0·54; 95 % CI: 0·30, 1·00; *P* = 0·05) and this finding was NS after adjusting for lactation status (OR per 5 % energy increment 0·55; 95 % CI: 0·30, 1·01; *P* = 0·06). Although not statistically significant, a greater percentage energy intake from barley was related to lower odds of overweight/obesity (OR per 5 % energy increment 0·85; 95 % CI: 0·72, 1·01; *P* = 0·06).

**Table 4:** OR (95 % CI) for percentage of total energy intake from top ten consumed protein foods in relation to prevalence of underweight and overweight/obesity among 1624 adults in Addis Ababa, Ethiopia

Protein source†	Mean % energy intake	Overweight/obese (*n* 1476)‡		Underweight (*n* 1123) §	
Teff	42·71				
Age- and sex-adjusted||		0·98	0·94, 1·02	1·02	0·96, 1·08
Multivariable-adjusted¶		1·01	0·97, 1·06	1·00	0·94, 1·06
Beef	6·11				
Age- and sex-adjusted		1·07	0·98, 1·16	0·94	0·82, 1·07
Multivariable-adjusted		0·97	0·89, 1·06	0·99	0·86, 1·14
Peas	5·88				
Age- and sex-adjusted		0·98	0·89, 1·07	1·06	0·92, 1·21
Multivariable-adjusted		1·05	0·95, 1·16	1·02	0·89, 1·17
Lentils	6·22				
Age- and sex-adjusted		1·00	0·92, 1·10	1·00	0·87, 1·15
Multivariable-adjusted		0·98	0·90, 1·08	0·98	0·85, 1·13
Wheat	5·46				
Age- and sex-adjusted		1·05	0·97, 1·15	0·90	0·78, 1·04
Multivariable-adjusted		1·05	0·97, 1·15	0·89	0·77, 1·02
Beans	2·83				
Age- and sex-adjusted		0·95	0·86, 1·05	1·07	0·92, 1·24
Multivariable-adjusted		0·97	0·88, 1·08	1·06	0·91, 1·24
Milk	1·11				
Age- and sex-adjusted		1·07	0·80, 1·43	0·50*	0·28, 0·91
Multivariable-adjusted		0·82	0·60, 1·12	0·54*	0·30, 1·00
Rice	2·95				
Age- and sex-adjusted		1·00	0·88, 1·13	1·06	0·88, 1·28
Multivariable-adjusted		0·96	0·84, 1·09	1·10	0·91, 1·33
Barley	1·14				
Age- and sex-adjusted		0·86	0·73, 1·01	0·72	0·50, 1·03
Multivariable-adjusted		0·85	0·72, 1·01	0·73	0·50, 1·05
Chickpeas	0·62				
Age- and sex-adjusted		0·86	0·61, 1·23	1·35	0·84, 2·18
Multivariable-adjusted		0·89	0·62, 1·29	1·44	0·89, 2·34

* *P* < 0·05.

† Protein source foods were included in models as percentage of total energy intake from each food. Models are examining a 5 % increase in percentage of total energy intake of each food.

‡ Overweight/obese is defined as BMI ≥ 25. The overweight/obese analysis was conducted among 1476 healthy weight, overweight and obese participants. 148 underweight participants were excluded from this analysis.

§ Underweight is defined as BMI < 18·5. The underweight analysis was conducted among 1123 healthy weight and underweight participants. 501 overweight and obese participants were excluded from this analysis.

|| Age- and sex-adjusted model is adjusted for female’s age (years, continuous) and sex (female, male)

¶ Multivariable-adjusted model is adjusted for female’s age, sex, female’s educational attainment (never attended school, completed primary or less, completed higher than primary), if female is married or living with partner (yes, no), wealth index (poorest, poor, wealthy, wealthiest), female’s religion (orthodox Christian or not orthodox Christian), household size (continuous) and female employed (yes, no).

For linear models with BMI as an outcome, results from secondary analyses adjusting for total energy intake (see online supplementary material, Supplemental Table 2) and excluding overweight participants (*n* 501) (see online supplementary material, Supplemental Table 3) were largely consistent with the primary analyses. When excluding all underweight participants (*n* 148), total protein was weakly associated with lower BMI when substituted for carbohydrate comparing the third and lowest quartiles of total protein intake (*β* –0·55; 95 % CI: –1·08, –0·01; *P* = 0·05), but not when comparing the highest and lowest quartiles (*β* –0·07; 95 % CI: –0·19, 0·04; *P* = 0·22) (see online supplementary material, Supplemental Table 4). Animal protein intake was related to lower BMI when substituted for plant protein in analyses excluding underweight participants, but this association was marginally significant (*β* per 1 % energy increment –0·11; 95 % CI: –0·23, 0·00; *P* = 0·05; *P*_trend_ across Q1–Q4 = 0·04).

In secondary analyses adjusting for total energy intake with overweight/obesity as an outcome, the association between total protein and overweight/obesity was NS in the fully adjusted carbohydrate substitution model (OR per 1 % energy increment: 0·93; 95 % CI: 0·87, 1·00; *P* = 0·06) (see online supplementary material, Supplemental Table 5). However, when adjusting for total energy intake, greater percentage energy from animal protein was still associated with lower odds of overweight/obesity in fully adjusted models substituting animal protein for carbohydrate (OR per 1 % energy increment: 0·90; 95 % CI: 0·83, 0·98; *P* = 0·01) and for plant protein (OR per 1 % energy increment: 0·91; 95 % CI: 0·85, 0·98; *P* = 0·01).

Results from secondary analyses using multivariable-adjusted energy partition models to examine substitution effects, which did not control for total energy intake, were consistent with the primary analyses. Among all participants, substituting fat for carbohydrate was associated with higher BMI, but substituting protein for carbohydrate was not. Among overweight/obese and healthy weight participants, substituting percentage energy from fat for carbohydrate was associated with higher odds of overweight/obesity, while substitution of protein for carbohydrate and protein for fat was associated with lower odds of obesity (see online supplementary material, Supplemental Table 6).

## Discussion

In this cross-sectional analysis among 1624 adults in Addis Ababa, Ethiopia, we found that percentage energy intake from total protein and animal protein were both significantly associated with lower odds of overweight/obesity when substituted for carbohydrate, especially among women. This study population had a very high intake of carbohydrates and very low intake of animal protein, which is consistent with other studies in low-income countries^(8)^. In addition, one-third of the study population was overweight or obese (34 % of women and 25 % of men), which is similar to previous estimates in Addis Ababa^(2,7)^.

To our knowledge, this is the first study in SSA to examine the associations between total, animal and plant protein intake in relation to underweight and overweight/obesity. Our findings are consistent with some previous studies in Western and Asian populations that have found that increased total protein as well as animal protein were associated with improved waist circumference^(18)^ and body composition^(17)^. Some randomised trials comparing higher protein diets to lower protein diets have reported decreases in body weight^(11,37–40)^ and greater fat loss with higher protein diets^(11,38,41)^. However, other trials have found that higher protein diets did not result in increased weight loss compared with other diets, especially over the long term^(41–43)^.A meta-analysis of thirty-two trials with greater than 12 months of follow up found that although higher protein diets showed benefits for weight loss in the short term, these benefits persisted to a smaller degree in the long term. However, greater benefits were observed long term in those with better compliance to higher-protein diets^(44)^.

Although additional evidence is needed from long-term trials, existing evidence from shorter-term trials suggests that higher-protein diets may have beneficial metabolic effects when protein is used to partially replace carbohydrates (especially carbohydrates from refined sources)^(41)^. Replacement of carbohydrate with protein may promote diet-induced thermogenesis, increase satiety which can lead to reduced subsequent energy intake and lower glycaemic load^(41,45)^. Protein intake may also protect against loss of lean body mass^(14)^. In the Ethiopian context, increasing protein intake in place of carbohydrate may be beneficial because carbohydrate intake is very high and food sources of carbohydrates are often refined^(7)^. In our sample, 69 % of calories were derived from carbohydrates and the major sources were teff (a cereal common in Ethiopia), refined wheat, maize and pasta.

Interestingly, we also found percentage energy from animal protein was associated with lower odds of overweight/obesity when substituted for plant protein, which conflicts with some previous studies in Western populations that have found beneficial effects of plant protein and harmful effects of animal protein on waist circumference, BMI and metabolic syndrome^(19–21)^. Food sources of animal protein tend to be higher in cholesterol, energy and saturated fats while food sources of plant protein could help to control body weight and improve body composition because of their associations with lower intakes of energy, total fat, cholesterol and saturated fat^(19)^. However, these findings and mechanisms in Western populations are likely not generalisable to low-income settings, especially to Ethiopia, where animal-source food consumption is much lower due to economic, cultural and religious factors and carbohydrate intake is very high^(46)^. The study populations in these Western studies were consuming 9–11 % of total energy from animal protein^(19,21)^, compared with 3 % of energy from animal protein in our Ethiopian study population. In addition, much of the protein intake in our study was from cereals (such as teff, wheat and rice) which may have less favourable effects on body weight than non-cereal plant proteins (such as pulses and nuts). A randomised controlled trial of protein intake and weight loss maintenance found that while substituting overall plant for animal protein was not associated with effects on body weight, a higher intake of cereal plant protein at the cost of non-cereal plant protein was associated with a larger increase in body weight^(47)^. Non-cereal plant protein sources such as pulses tend to have a lower glycaemic load and may increase satiety, compared with cereal protein sources (some of which may be refined carbohydrates)^(47)^. It is possible that in low-income settings with high intakes of carbohydrate and low consumption of animal-source foods, protein from animal sources may be beneficial in preventing overweight or obesity, given that much of the protein and energy in the diet comes from cereals.

A strength of this study is that we were able to approximate usual dietary intake through a non-quantitative FFQ adapted for use in Ethiopia. Most previous dietary surveys in Ethiopia have relied on a single 24-hour recall, which are not considered representative of usual dietary patterns. We also conducted a detailed assessment of confounders related to protein intake, overweight/obesity and underweight. Consistent with previous studies, we found a number of socio-demographic factors that were strongly related to protein intake including age, sex, religion, marital status, wealth and household food insecurity^(48)^. It is well-documented that these confounders are also strongly associated with obesity risk in sub-Saharan Africa^(5,48)^.

FFQs are designed primarily to generate estimates of relative intake, which gives us the ability to distinguish between high and low consumers of a given nutrient, but they can only approximate absolute intake and are subject to additional limitations including recall and self-report biases. We were able to use a non-quantitative FFQ in this study because our analyses did not rely on absolute intakes. While it would have been ideal to use an FFQ validated in an Ethiopian population, no such instrument exists to our knowledge; therefore, we adapted this FFQ from one validated in Tanzania. Additionally, we were unable to capture seasonal variations in food intake because this FFQ was administered at a single time point. During the time period of the survey (Jan–Feb), urban energy intake tends to be higher than during the lean season in June and July^(49)^. Despite this, we found that a large proportion of our sample were below the estimated average requirement for protein intake (21 % of men, 23 % of non-lactating women and 62 % of lactating women). However, these findings should be interpreted with caution because the FFQ was not validated in this population and the low levels of protein intake we found are in part due to the low total energy intake in this sample, which may have been under-reported. That said, levels of intake reported by our study participants were consistent with previous estimates from Ethiopia^(7)^.

An additional limitation of this study is possible residual confounding from measured and unmeasured factors, such as physical activity. We also lack some key covariates for the men in the sample (including age), but were able to use the female’s covariates as the male and female participants were living in the same household and in most cases were married. However, it is possible that age, education and employment of the female participant could be different than that of the male participant, which could lead to bias in the results of the adjusted models for the male participants. There is also potential for selection bias, since the study only included households who consented to participate in the survey and contained young children, which may not be representative of the overall population of Addis Ababa. However, this survey had a very high response rate (97 %) and the characteristics of the study population are similar to those of the greater urban Ethiopian population in terms of age, educational attainment and household size^(2)^. In addition, Ethiopia has a high fertility rate and a long median duration of breastfeeding (2 years) which may also partially explain the high proportion of lactating women in this sample^(2)^. To further address these limitations, we adjusted for socio-demographic and household characteristics in all analyses and adjusted for lactation status in secondary analyses. Lastly, this study focused on Addis Ababa, a major city and results may not be generalisable to rural areas of Ethiopia or other SSA contexts, where dietary patterns and prevalence of overweight differ. In Addis Ababa, consumption of animal source foods is twice as high as in rural areas of Ethiopia^(50)^ and overweight/obesity prevalence is six times higher among women and thirteen times higher among men in urban compared with rural areas of Ethiopia^(2)^.

The cross-sectional design of this study is an additional limitation as it restricts our ability to make causal inferences and poses unique challenges when studying bodyweight as an outcome because participants may change their diets due to awareness of their weight. Repeated assessments of changes over time in dietary factors and body weight are needed to understand long-term effects of protein intake on overweight/obesity and underweight^(35)^. The cross-sectional design could explain why we did not observe any associations between underweight and animal or plant protein intake.

Animal protein has traditionally been thought of as beneficial in the context of undernutrition, as it provides higher-quality protein (defined by the amino acid content) than most plant sources, along with other essential nutrients^(8,15)^. Although animal-source foods may be valuable in preventing and treating undernutrition, promotion of animal-source foods, especially meat, is controversial as they have greater environmental impact than plant protein sources^(51)^. One study in Addis Ababa found that meeting adult and child recommended daily protein intakes with plant-based foods would have a lower environmental impact than meeting this gap with animal-based foods, but would still result in an estimated 65 % increase in land and water use and a 2 % increase in greenhouse gas emissions. Meeting this protein gap with a milk and red meat strategy would result in an estimated 190 % increase in land and water use and 257 % increase in greenhouse gas emissions^(10)^.

Despite their environmental impacts, animal-source foods are a valuable source of protein and micronutrients and providing some animal-source foods alongside plant-based foods may offer additional health benefits in the Ethiopian context^(10)^. In Africa, animal-source food production can be sustainable through using grassland which cannot be used for crop production and by converting inedible crop by-products into edible food. It can also aid in reducing fertiliser use, cycling nutrients within the ecosystem and is an important mechanism to diversify farmers’ income in the case crop production is reduced^(52–54)^. The Ethiopian Government has demonstrated their commitment to transitioning traditional livestock practices to climate-smart practices through policy interventions as outlined in their Livestock Master Plan and Climate Resilience and Green Economy Strategy^(54)^.

For optimal human and planetary health, the EAT-Lancet Commission has recommended adopting a global reference diet, consisting of mainly plant-source foods (vegetables, fruits, whole grains, and legumes and nuts), and limited or modest amounts of animal-source foods (such as red or processed meat)^(15)^. However, in our sample, animal-source protein intake was only 11 g per day, which is less than half of the world average of animal-source protein availability in 2011 and much less than the amounts in the EAT-Lancet healthy reference diet^(15)^. In Ethiopia, animal protein consumption tends to be limited due to religious and social norms and the availability, accessibility and affordability of animal-source foods^(55)^. It is estimated that to achieve global dietary recommendations in SSA, increases in food consumption-related greenhouse gas emissions would be necessary, but that these small increases would be far outweighed by higher-income countries adopting diets lower in meat consumption^(51)^.

## Conclusion

Innovative strategies are needed to combat the complex, coexisting problems of over and undernutrition in SSA. Our results suggest that in the context of diets that are very high in carbohydrate such as those in Ethiopia, a greater proportion of energy intake from protein may be associated with lower prevalence of overweight and obesity. However, due to the cross sectional design of this study and limited generalisability to other contexts, our findings should be interpreted with caution. Community-based longitudinal data is scarce in Ethiopia, but such studies are needed to further clarify associations between dietary intake with underweight and overweight/obesity, track changes over time and account for seasonality and urban/rural differences in consumption. The majority of the literature on dietary protein and bodyweight is from Western settings and is likely not generalisable to SSA, where dietary patterns are very different. Longitudinal dietary surveys in Ethiopia are needed to inform local guidelines, policies and programmes to combat the double burden of malnutrition. Given the constraints limiting animal protein consumption in Ethiopia, future research should also assess the feasibility of incorporating modest amounts of animal products into Ethiopian diets.

## Supplementary material

For supplementary material accompanying this paper visit https://doi.org/10.1017/S1368980022001100. click here to view supplementary material

## References

[1] Institute for Health Metrics and Evaluation (IHME) (2018) Ethiopia Profile. Seattle, WA: IHME.

[2] Central Statistical Agency – CSA/Ethiopia & ICF (2017) Ethiopia Demographic and Health Survey 2016. Addis Ababa, Ethiopia: CSA and ICF.

[3] WHO (2011) Global Status Report on Noncommunicable Diseases 2010. Geneva: WHO.

[4] Wells JC , Sawaya AL , Wibaek R et al. (2020) The double burden of malnutrition: aetiological pathways and consequences for health. Lancet 395, 75–88. 31852605 10.1016/S0140-6736(19)32472-9 PMC7613491

[5] Tebekaw Y , Teller C & Colón-Ramos U (2014) The burden of underweight and overweight among women in Addis Ababa, Ethiopia. BMC Public Health 14, 1126. 25361603 10.1186/1471-2458-14-1126 PMC4228094

[6] Misganaw A , Melaku YA , Tessema GA et al. (2017) National disability-adjusted life years (DALYs) for 257 diseases and injuries in Ethiopia, 1990–2015: findings from the global burden of disease study 2015. Popul Health Metr 15, 28. 28732542 10.1186/s12963-017-0146-0 PMC5521136

[7] Ethiopian Public Health Institute (2013) Ethiopia National Food Consumption Survey; available at https://ephi.gov.et/ (accessed January 2021).

[8] Schönfeldt HC & Gibson Hall N (2012) Dietary protein quality and malnutrition in Africa. Br J Nutr 108, S69–S76. 23107550 10.1017/S0007114512002553

[9] Ghosh S , Suri D & Uauy R (2012) Assessment of protein adequacy in developing countries: quality matters. Br J Nutr 108, S77–S87. 23107551 10.1017/S0007114512002577

[10] Blakstad MM , Danaei G , Tadesse AW et al. (2021) Life expectancy and agricultural environmental impacts in Addis Ababa can be improved through optimized plant and animal protein consumption. Nat Food 2, 291–298. 37118473 10.1038/s43016-021-00264-2

[11] Green KK , Shea JL , Vasdev S et al. (2010) Higher dietary protein intake is associated with lower body fat in the Newfoundland population. Clin Med Insights Endocrinol Diabetes 3, 25–35. 22879784 10.4137/cmed.s4619 PMC3411511

[12] Pasiakos SM , Lieberman HR & Fulgoni VL (2015) Higher-protein diets are associated with higher HDL cholesterol and lower BMI and waist circumference in US adults. J Nutr 145, 605–614. 25733478 10.3945/jn.114.205203

[13] Wycherley TP , Moran LJ , Clifton PM et al. (2012) Effects of energy-restricted high-protein, low-fat compared with standard-protein, low-fat diets: a meta-analysis of randomized controlled trials. Am J Clin Nutr 96, 1281–1298. 23097268 10.3945/ajcn.112.044321

[14] Liu AY , Silvestre MP & Poppitt SD (2015) Prevention of type 2 diabetes through lifestyle modification: is there a role for higher-protein diets? Adv Nutr 6, 665–673. 26567192 10.3945/an.115.008821 PMC4642418

[15] Willett W , Rockström J , Loken B et al. (2019) Food in the anthropocene: the EAT-Lancet commission on healthy diets from sustainable food systems. Lancet 393, 447–492. 30660336 10.1016/S0140-6736(18)31788-4

[16] Krasevec J , An X , Kumapley R et al. (2017) Diet quality and risk of stunting among infants and young children in low- and middle-income countries. Matern Child Nutr 13, e12430. 29032628 10.1111/mcn.12430 PMC6865990

[17] Berryman CE , Agarwal S , Lieberman HR et al. (2016) Diets higher in animal and plant protein are associated with lower adiposity and do not impair kidney function in US adults. Am J Clin Nutr 104, 743–749. 27465374 10.3945/ajcn.116.133819

[18] Park KB , Park HA , Kang JH et al. (2018) Animal and plant protein intake and body mass index and waist circumference in a korean elderly population. Nutrients 10, 577. 29738479 10.3390/nu10050577 PMC5986457

[19] Lin Y , Bolca S , Vandevijvere S et al. (2011) Plant and animal protein intake and its association with overweight and obesity among the Belgian population. Br J Nutr 105, 1106–1116. 21144092 10.1017/S0007114510004642

[20] Hruby A & Jacques PF (2018) Dietary protein and changes in markers of cardiometabolic health across 20 years of follow-up in middle-aged Americans. Public Health Nutr 21, 2998–3010. 30115136 10.1017/S1368980018001854 PMC6185746

[21] Shang X , Scott D , Hodge A et al. (2017) Dietary protein from different food sources, incident metabolic syndrome and changes in its components: an 11-year longitudinal study in healthy community-dwelling adults. Clin Nutr 36, 1540–1548. 27746001 10.1016/j.clnu.2016.09.024

[22] Song M , Fung TT , Hu FB et al. (2016) Association of animal and plant protein intake with all-cause and cause-specific mortality. JAMA Intern Med 176, 1453–1463. 27479196 10.1001/jamainternmed.2016.4182 PMC5048552

[23] International Food Policy Research Institute (2017) 2017 Global Food Policy Report. Washington, DC: International Food Policy Research Institute.

[24] Bekele TH , de Vries JJHM , Trijsburg L et al. (2019) Methodology for developing and evaluating food-based dietary guidelines and a Healthy Eating Index for Ethiopia: a study protocol. BMJ Open 9, e027846. 10.1136/bmjopen-2018-027846 PMC6661676 31315863

[25] Zack RM , Irema K , Kazonda P et al. (2018) Validity of an FFQ to measure nutrient and food intakes in Tanzania. Public Health Nutr 21, 2211–2220. 29656731 10.1017/S1368980018000848 PMC6101256

[26] Ethiopian Health and Nutrition Research Institute (EHNRI) (1998) Food Composition Table for Use in Ethiopia. Addis Ababa, Ethiopia: EHNRI.

[27] Tadesse AW , Hemler EC , Andersen C et al. (2019) Anemia prevalence and etiology among women, men, and children in Ethiopia: a study protocol for a national population-based survey. BMC Public Health 19, 1369. 31651278 10.1186/s12889-019-7647-7 PMC6814127

[28] Lukmanji Z , Hertzmark E , Mlingi N et al. (2008) Tanzania Food Composition Tables. Dar es Salaam, Tanzania: Muhimbili University of Health and Allied Sciences, Tanzania Food and Nutrition Centre and Harvard School of Public Health.

[29] Institute of Medicine (2005) Dietary Reference Intakes for Energy, Carbohydrate, Fiber, Fat, Fatty Acids, Cholesterol, Protein, and Amino Acids. Washington, DC: Institute of Medicine. 10.1016/s0002-8223(02)90346-9 12449285

[30] Vyas S & Kumaranayake L (2006) Constructing socio-economic status indices: how to use principal components analysis. Health Policy Plann 21, 459–468. 10.1093/heapol/czl029 17030551

[31] Coates J , Swindale A & Bilinsky P (2007) Household Food Insecurity Access Scale (HFIAS) for Measurement of Household Food Access: Indicator Guide (v.3). Food and Nutrition Technical Assistance Project. Washington, D.C.: Academy for Educational Development.

[32] Hoque ME , Hasan MT , Rahman M et al. (2017) Double burden of underweight and overweight among Bangladeshi adults differs between men and women: evidence from a nationally representative survey. Public Health Nutr 20, 2183–2191. 28633684 10.1017/S1368980017000957 PMC10261637

[33] Finucane MM , Stevens GA , Cowan MJ et al. (2011) National, regional, and global trends in body-mass index since 1980: systematic analysis of health examination surveys and epidemiological studies with 960 country-years and 9·1 million participants. Lancet 377, 557–567. 21295846 10.1016/S0140-6736(10)62037-5 PMC4472365

[34] Willett WC (1990) Nutritional Epidemiology. New York: Oxford University Press.

[35] Mozaffarian D , Hao T , Rimm EB et al. (2011) Changes in diet and lifestyle and long-term weight gain in women and men. N Engl J Med 364, 2392–2404. 21696306 10.1056/NEJMoa1014296 PMC3151731

[36] Westerterp-Plantenga MS , Lemmens SG & Westerterp KR (2012) Dietary protein – its role in satiety, energetics, weight loss and health. Br J Nutr 108, S105–S112. 23107521 10.1017/S0007114512002589

[37] Foster GD , Wyatt HR , Hill JO et al. (2003) A randomized trial of a low-carbohydrate diet for obesity. N Engl J Med 348, 2082–2090. 12761365 10.1056/NEJMoa022207

[38] Samaha FF , Iqbal N , Seshadri P et al. (2003) A low-carbohydrate as compared with a low-fat diet in severe obesity. N Engl J Med 348, 2074–2081. 12761364 10.1056/NEJMoa022637

[39] Skov AR , Toubro S , Rønn B et al. (1999) Randomized trial on protein *v.* carbohydrate in ad libitum fat reduced diet for the treatment of obesity. Int J Obes Relat Metab Disord 23, 528–536. 10375057 10.1038/sj.ijo.0800867

[40] Dong JY , Zhang ZL , Wang PY et al. (2013) Effects of high-protein diets on body weight, glycaemic control, blood lipids and blood pressure in type 2 diabetes: meta-analysis of randomised controlled trials. Br J Nutr 110, 781–789. 23829939 10.1017/S0007114513002055

[41] Halton TL & Hu FB (2004) The effects of high protein diets on thermogenesis, satiety and weight loss: a critical review. J Am Coll Nutr 23, 373–385. 15466943 10.1080/07315724.2004.10719381

[42] Sacks FM , Bray GA , Carey VJ et al. (2009) Comparison of weight-loss diets with different compositions of fat, protein, and carbohydrates. N Engl J Med 360, 859–873. 19246357 10.1056/NEJMoa0804748 PMC2763382

[43] Noakes M , Keogh JB , Foster PR et al. (2005) Effect of an energy-restricted, high-protein, low-fat diet relative to a conventional high-carbohydrate, low-fat diet on weight loss, body composition, nutritional status, and markers of cardiovascular health in obese women. Am J Clin Nutr 81, 1298–1306. 15941879 10.1093/ajcn/81.6.1298

[44] Clifton PM , Condo D & Keogh JB (2014) Long term weight maintenance after advice to consume low carbohydrate, higher protein diets – a systematic review and meta analysis. Nutr Metab Cardiovasc Dis 24, 224–235. 24472635 10.1016/j.numecd.2013.11.006

[45] Weigle DS , Breen PA , Matthys CC et al. (2005) A high-protein diet induces sustained reductions in appetite, ad libitum caloric intake, and body weight despite compensatory changes in diurnal plasma leptin and ghrelin concentrations. Am J Clin Nutr 82, 41–48. 16002798 10.1093/ajcn.82.1.41

[46] Zellelew TB (2014) Meat abstinence and its positive environmental effect: examining the fasting etiquettes of the Ethiopian Orthodox Church. Crit Res Religion 2, 134–146.

[47] van Baak MA , Larsen TM , Jebb SA et al. (2017) Dietary intake of protein from different sources and weight regain, changes in body composition and cardiometabolic risk factors after weight loss: the DIOGenes study. Nutrients 9, 1326. 29211027 10.3390/nu9121326 PMC5748776

[48] Steyn NP & McHiza ZJ (2014) Obesity and the nutrition transition in Sub-Saharan Africa. Ann N Y Acad Sci 1311, 88–101. 24725148 10.1111/nyas.12433

[49] Hirvonen K , Taffesse AS & Worku Hassen I (2016) Seasonality and household diets in Ethiopia. Public Health Nutr 19, 1723–1730. 26585676 10.1017/S1368980015003237 PMC10271090

[50] Abegaz GA , Hassen IW & Minten B (2018) Consumption of Animal-Source Foods in Ethiopia: Patterns, Changes, and Determinants. ESSP Working Paper 113. Washington, DC; Addis Ababa, Ethiopia: International Food Policy Research Institute (IFPRI) and Ethiopian Development Research Institute (EDRI).

[51] Springmann M , Godfray HC , Rayner M et al. (2016) Analysis and valuation of the health and climate change cobenefits of dietary change. Proc Natl Acad Sci USA 113, 4146–4151. 27001851 10.1073/pnas.1523119113 PMC4839446

[52] Kim BF , Santo RE , Scatterday AP et al. (2020) Country-specific dietary shifts to mitigate climate and water crises. Global Environ Change 62, 101926.

[53] Mehrabi Z , Gill M , van Wijk M et al. (2020) Livestock policy for sustainable development. Nat Food 1, 160–165.

[54] Federal Democratic Republic of Ethiopia (2021) Updated Nationally Determined Contribution. United Nations Framework Convention on Climate Change; available at https://www4.unfccc.int/sites/ndcstaging/PublishedDocuments/Ethiopia%20First/Ethiopia%27s%20updated%20NDC%20JULY%202021%20Submission_.pdf (accessed March 2021).

[55] Bliznashka L , Passarelli S , Canavan CR et al. (2021) Changes and challenges in markets for animal source foods: a qualitative study among market vendors in Addis Ababa, Ethiopia. Food Secur 13, 583–595.

