# Supplementary material for: Associations of percentage energy intake from total, animal and plant protein with overweight/obesity and underweight among adults in Addis Ababa, Ethiopia
Source: Public Health Nutr. 2022 May 16;25(11):3107–20. doi: 10.1017/S1368980022001100 (PMC9991810; doi:10.1017/S1368980022001100)
Supplement: Supplementary file 1 [file S1368980022001100sup.zip › S1368980022001100sup003.docx]

**Associations of percent energy intake from total, animal and plant protein with overweight/obesity and underweight among adults in Addis Ababa, Ethiopia**

**Online Supplementary Material**

**Supplementary Table 1.** Sex-specific odds ratios (95% confidence intervals) for percentage of energy intake from total protein, plant protein and animal protein in relation to prevalence of underweight or overweight/obesity among 1,624 women and men in Addis Ababa, Ethiopia

| Percent Energy from Total Protein^1^ | | | | | | | | | | |
| --- | --- | --- | --- | --- | --- | --- | --- | --- | --- | --- |
|  | Women (n=992) | | | | | Men (n=632) | | | | |
|  | Q1 | Q2 | Q3 | Q4 | Per 1% difference | Q1 | Q2 | Q3 | Q4 | Per 1% difference |
| Overweight/Obese^2^ |  |  |  |  |  |  |  |  |  |  |
| Age-adjusted^3^ | 1.00 (Ref) | 1.20  (0.82, 1.75) | 0.94  (0.64, 1.39) | 1.10  (0.73, 1.66) | 0.98  (0.90, 1.06) | 1.00 (Ref) | 1.34  (0.77, 2.35) | 0.75  (0.42, 1.34) | 1.48  (0.89, 2.47) | 1.05  (0.95, 1.15) |
| Multi-variable adjusted carb^4^ | 1.00 (Ref) | 0.99  (0.67, 1.48) | 0.72  (0.48, 1.09) | 0.74  (0.47, 1.17) | 0.88*  (0.81, 0.97) | 1.00 (Ref) | 1.33  (0.73, 2.43) | 0.72  (0.38, 1.36) | 1.27  (0.68, 2.420 | 0.99  (0.88, 1.12) |
| Underweight^5^ |  |  |  |  |  |  |  |  |  |  |
| Age-adjusted | 1.00 (Ref) | 0.85  (0.47, 1.54) | 0.63  (0.34, 1.17) | 1.04  (0.56, 1.93) | 0.95  (0.83, 1.07) | 1.00 (Ref) | 0.71  (0.30, 1.64) | 0.52  (0.22, 1.23) | 0.90  (0.43, 1.87) | 0.98  (0.85, 1.14) |
| Multi-variable adjusted carb | 1.00 (Ref) | 0.85  (0.47, 1.56) | 0.63  (0.33, 1.20) | 0.95  (0.48, 1.88) | 0.92  (0.80, 1.06) | 1.00 (Ref) | 0.83  (0.34, 2.04) | 0.62  (0.25, 1.57) | 1.36  (0.55, 3.35) | 1.09  (0.90, 1.32) |
| Percent Energy from Plant Protein | | | | | | | |  |  |  |
| Overweight/Obese |  |  |  |  |  |  |  |  |  |  |
| Age-adjusted | 1.00 (Ref) | 0.86  (0.58, 1.27) | 1.09  (0.75, 1.60) | 0.95  (0.64, 1.41) | 0.98  (0.91, 1.06) | 1.00 (Ref) | 0.61  (0.37, 1.01) | 0.57*  (0.33, 0.97) | 0.55*  (0.33, 0.92) | 0.89*  (0.80, 0.98) |
| Multi-variable  adjusted carb | 1.00 (Ref) | 0.83  (0.54, 1.29) | 1.02  (0.66, 1.60) | 0.99  (0.60, 1.64) | 0.96  (0.87, 1.07) | 1.00 (Ref) | 0.66  (0.37, 1.16) | 0.70  (0.37, 1.31) | 0.80  (0.40, 1.57) | 0.97  (0.84, 1.13) |
| Multi-variable adjusted animal^6^ | 1.00 (Ref) | 1.06  (0.70, 1.61) | 1.48  (0.98, 2.23) | 1.71*  (1.08, 2.73) | 1.11*  (1.01, 1.22) | 1.00 (Ref) | 0.66  (0.38, 1.12) | 0.71  (0.40, 1.25 ) | 0.77  (0.43, 1.39) | 0.96  (0.85, 1.08) |
| Underweight |  |  |  |  |  |  |  |  |  |  |
| Age-adjusted | 1.00 (Ref) | 0.98  (0.52, 1.85) | 1.37  (0.76, 2.47) | 1.02  (0.54, 1.94) | 1.05  (0.93, 1.18) | 1.00 (Ref) | 1.17  (0.43, 3.14) | 1.93  (0.75, 4.97) | 1.97  (0.79, 4.91) | 1.15  (0.98, 1.34) |
| Multi-variable adjusted carb | 1.00 (Ref) | 0.90  (0.44, 1.83) | 1.09  (0.55, 2.17) | 0.76  (0.35, 1.67) | 0.97  (0.83, 1.14) | 1.00 (Ref) | 1.16  (0.39, 3.50) | 1.93  (0.64, 5.78) | 1.77  (0.55, 5.68) | 1.11  (0.90, 1.38) |
| Multi-variable  adjusted animal | 1.00 (Ref) | 1.11  (0.56, 2.21) | 1.44  (0.76, 2.73) | 1.15  (0.55, 2.42) | 1.07  (0.93, 1.23) | 1.00 (Ref) | 1.06  (0.37, 3.04) | 1.70  (0.63, 4.60) | 1.53  (0.56, 4.20) | 1.09  (0.91, 1.32) |
| Percent Energy from Animal Protein | | | | | | | | | | |
| Overweight/Obese |  |  |  |  |  |  |  |  |  |  |
| Age-adjusted | 1.00 (Ref) | 1.38  (0.93, 2.04) | 1.24  (0.83, 1.84) | 1.00  (0.66, 1.52) | 1.00  (0.94, 1.06) | 1.00 (Ref) | 1.40  (0.80, 2.45) | 1.44  (0.83, 2.49) | 1.87*  (1.13, 3.11)* | 1.09*  (1.01, 1.17) |
| Multi-variable adjusted carb | 1.00 (Ref) | 0.88  (0.57, 1.36) | 0.58*  (0.35, 0.94) | 0.32**  (0.17, 0.59) | 0.83**  (0.75, 0.92) | 1.00 (Ref) | 1.29  (0.70, 2.37) | 1.08  (0.56, 2.08) | 1.10  (0.50, 2.42) | 1.01  (0.88, 1.15) |
| Multi-variable adjusted plant^7^ | 1.00 (Ref) | 0.90  (0.59, 1.40) | 0.61*  (0.38, 1.00) | 0.36**  (0.20, 0.66) | 0.85**  (0.77, 0.93) | 1.00 (Ref) | 1.32  (0.72, 2.41) | 1.10  (0.58, 2.09) | 1.11  (0.53, 2.29) | 1.01  (0.90, 1.13) |
| Underweight |  |  |  |  |  |  |  |  |  |  |
| Age-adjusted | 1.00 (Ref) | 1.04  (0.58, 1.87) | 0.96  (0.52, 1.76) | 0.69  (0.36, 1.34) | 0.94  (0.84, 1.04) | 1.00 (Ref) | 0.70  (0.31, 1.59) | 0.72  (0.33, 1.59) | 0.72  (0.33, 1.55) | 0.91  (0.80, 1.04) |
| Multi-variable adjusted carb | 1.00 (Ref) | 0.92  (0.49, 1.75) | 0.76  (0.37, 1.58) | 0.52  (0.20, 1.35) | 0.87  (0.73, 1.02) | 1.00 (Ref) | 0.94  (0.38, 2.28) | 1.35  (0.52, 3.53) | 2.11  (0.62, 7.13) | 1.05  (0.84, 1.32) |
| Multi-variable adjusted plant | 1.00 (Ref) | 0.94  (0.50, 1.78) | 0.79  (0.39, 1.62) | 0.57  (0.23, 1.38) | 0.89  (0.77, 1.03) | 1.00 (Ref) | 0.87  (0.36, 2.10) | 1.15  (0.45, 2.92) | 1.47  (0.47, 4.57) | 0.99  (0.81, 1.20) |

*P<.05

**P< 0.001

1. Percent energy intake from total protein, plant protein and animal protein were examined in logistic regression models as continuous exposures and also in quartiles; Q1 represents the lowest percent energy intake from total, plant or animal protein.

2. Overweight is defined as BMI ≥ 25 and is compared with a reference category of non-overweight/obese (BMI < 25). All underweight participants (n=96 for women; n=52 for men) were excluded from the overweight analysis.

3. Adjusted for female's age (years, continuous)

4. Multi-variable adjusted carbohydrate substitution model is adjusted for female's age (years), percentage of calories from total fat (continuous), female’s educational attainment (never attended school, completed primary or less, completed higher than primary) , if female is married or living with partner (yes, no), wealth index (poorest, poor, wealthy, wealthiest), female’s religion (orthodox Christian or not orthodox Christian), household size (continuous), female employed (yes, no). For plant protein, the model is additionally adjusted for percentage of calories from animal protein. For animal protein, it is additionally adjusted for percentage of calories from plant protein.

5. Underweight is defined as BMI < 18.5 and is compared with a reference category of non-underweight (BMI ≥ 18.5). All overweight and obese participants (n=341 for women; n=160 for men) were excluded from the underweight analysis.

6. Multi-variable adjusted animal protein substitution model is adjusted for female's age (years), percentage of calories from total fat (continuous), percentage of calories from total carbohydrate (continuous), female’s educational attainment (never attended school, completed primary or less, completed higher than primary) , if female is married or living with partner (yes, no), wealth index (poorest, poor, wealthy, wealthiest), female’s religion (orthodox Christian or not orthodox Christian), household size (continuous), female employed (yes, no).

7. Multi-variable adjusted plant protein substitution model is adjusted for the same covariates as the multi-variable animal protein substitution model.

**Supplementary Table 2.** Beta coefficients (95% confidence intervals) for associations of total energy intake and body mass index (BMI); and percent energy intake from total, plant and animal protein in relation to BMI adjusted for total energy intake among 1,624 adults in Addis Ababa, Ethiopia

|  | Q1 | Q2 | Q3 | Q4 | Per 100 kcal increment | P_trend_ |
| --- | --- | --- | --- | --- | --- | --- |
| Total Energy Intake | | | | | | |
| Age- and sex-adjusted^1^ | 0.0 (Ref) | 0.36  (-0.18, 0.90) | 0.27  (-0.27, 0.82) | 0.36  (-0.18, 0.91) | 0.00  (-0.03, 0.04) | 0.26 |
| Multivariable-adjusted^2^ | 0.0 (Ref) | 0.09  (-0.44, 0.63) | -0.07  (-0.61, 0.47) | -0.11  (-0.66, 0.44) | -0.02  (-0.06, 0.01) | 0.58 |
| Percent Energy from Total Protein | | | | | | |
|  | Q1 | Q2 | Q3 | Q4 | Per 1% difference^3^ | P_trend_ |
| Age, sex and energy-adjusted^4^ | 0.0 (Ref) | 0.40  (-0.16, 0.95) | 0.08  (-0.49, 0.64) | 0.63*  (0.06, 1.21) | 0.09  (-0.02, 0.21) | 0.07 |
| Multivariable energy-adjusted^5^ | 0.0 (Ref) | 0.32  (-0.23, 0.87) | -0.06  (-0.61, 0.50) | 0.37  (-0.20, 0.94) | 0.03  (-0.08, 0.14) | 0.37 |
| Multivariable energy-adjusted carbohydrate substitution^6^ | 0.0 (Ref) | 0.23  (-0.32, 0.78) | -0.19  (-0.75, 0.38) | 0.09  (-0.51, 0.70) | -0.04  (-0.16, 0.09) | 0.94 |
| Percent Energy from Plant Protein | | | | | | |
| Age, sex and energy-adjusted | 0.0 (Ref) | -0.47 (-1.01, 0.07) | -0.43  (-0.97, 0.12) | -0.71* (-1.25, -0.17) | -0.17* (-0.27, -0.06) | 0.01 |
| Multivariable energy-adjusted | 0.0 (Ref) | -0.27  (-0.81, 0.26) | 0.02  (-0.52, 0.57) | -0.02 (-0.58, 0.54) | -0.02 (-0.13, 0.09) | 0.85 |
| Multivariable energy-adjusted carbohydrate substitution | 0.0 (Ref) | -0.13 (-0.70, 0.45) | 0.15 (-0.48, 0.77) | 0.12 (-0.55, 0.80) | 0.00  (-0.14, 0.14) | 0.56 |
| Multivariable energy- adjusted animal protein substitution^7^ | 0.0 (Ref) | -0.06 (-0.61, 0.49) | 0.25 (-0.32, 0.82) | 0.26 (-0.35, 0.88) | 0.04 (-0.08, 0.16) | 0.27 |
| Percent Energy from Animal Protein | | | | | | |
| Mean % energy | 0.0 | 1.2 | 3.0 | 5.7 | 2.5 |  |
| Age, sex and energy-adjusted | 0.0 (Ref) | 0.91* (0.35, 1.47) | 0.78* (0.21, 1.36) | 1.11** (0.54, 1.67) | 0.16** (0.08, 0.24) | < 0.01 |
| Multivariable energy- adjusted | 0.0 (Ref) | 0.58*  (0.02, 1.14) | 0.25  (-0.34, 0.83) | 0.26  (-0.33, 0.86) | 0.03  (-0.05, 0.12) | 0.85 |
| Multivariable energy- adjusted carbohydrate substitution | 0.0 (Ref) | 0.41 (-0.16, 0.98) | -0.11 (-0.75, 0.54) | -0.38 (-1.17, 0.41) | -0.06 (-0.20, 0.07) | 0.15 |
| Multivariable energy-adjusted plant protein substitution^7^ | 0.0 (Ref) | 0.40 (-0.16, 0.97) | -0.12 (-0.74, 0.51) | -0.40 (-1.13, 0.33) | -0.08  (-0.19, 0.04) | 0.09 |

* P<.05

** P < .001

1. The age- and sex-adjusted model is adjusted for female's age (years, continuous) and sex (female, male)

2. The multivariable-adjusted model is adjusted for female's age, sex, female’s educational attainment (never attended school, completed primary or less, completed higher than primary) , if female is married or living with partner (yes, no), wealth index (poorest, poor, wealthy, wealthiest), female’s religion (orthodox Christian or not orthodox Christian), household size (continuous) and female employed (yes, no).

3. Beta coefficients are in kg/m^2^ per 1% difference in percent energy

4. The age, sex and energy-adjusted model is adjusted for female's age (years, continuous), sex (female, male) and total energy intake (kcal, continous)

5. The multivariable energy-adjusted model is the multivariable-adjusted model additionally adjusted for total energy intake

6. The multivariable energy-adjusted carbohydrate substitution model is the multivariable energy-adjusted model additionally adjusted for percentage of calories from total fat (continuous). When plant protein is the exposure, the model is additionally adjusted for percentage of calories from animal protein. When animal protein is the exposure, the model is additionally adjusted for percentage of calories from plant protein.

7. The multivariable energy-adjusted animal protein substitution model and the multivariable energy-adjusted plant protein substitution models are both adjusted for total energy intake, female's age, sex, percentage of calories from fat, percentage of calories from carbohydrate (continuous), female’s educational attainment, if female is married or living with partner, wealth index, female’s religion, household size and female employed.

**Supplementary Table 3.** Beta coefficients (95% confidence intervals) for percent energy intake from total protein, animal and plant protein in relation to body mass index (BMI) among 1,123 underweight and healthy weight adults^1^ in Addis Ababa, Ethiopia

|  | Q1 | Q2 | Q3 | Q4 | Per 1% difference^2^ | P_trend_ |
| --- | --- | --- | --- | --- | --- | --- |
| Percent Energy from Total Protein | | | | | | |
| Mean % energy | 11.9 | 13.6 | 14.8 | 16.5 | 14.2 |  |
| Age- and sex-adjusted^3^ | 0.0 (Ref) | 0.16  (-0.22, 0.54) | 0.30  (-0.07, 0.67) | 0.25  (-0.13, 0.63) | 0.07  (-0.00, 0.14) | 0.13 |
| Multivariable-adjusted^4^ | 0.0 (Ref) | 0.09  (-0.29, 0.46) | 0.19  (-0.18, 0.56) | 0.13  (-0.26, 0.51) | 0.04  (-0.03, 0.12) | 0.42 |
| Multivariable-adjusted carbohydrate substitution^5^ | 0.0 (Ref) | 0.10  (-0.29, 0.48) | 0.20  (-0.18, 0.59) | 0.15  (-0.27, 0.58) | 0.05  (-0.03, 0.14) | 0.39 |
| Percent Energy from Plant Protein | | | | | | |
| Mean % energy | 9.4 | 11.1 | 12.3 | 14.1 | 11.8 |  |
| Age- and sex-adjusted | 0.0 (Ref) | -0.27  (-0.65, 0.12) | -0.33 (-0.72, 0.05) | -0.37 (-0.75, 0.02) | -0.09* (-0.16, -0.01) | 0.07 |
| Multivariable-adjusted | 0.0 (Ref) | -0.19 (-0.57, 0.19) | -0.16 (-0.55, 0.22) | -0.05 (-0.45, 0.34) | -0.02 (-0.10, 0.06) | 0.88 |
| Multivariable-adjusted carbohydrate substitution | 0.0 (Ref) | -0.10 (-0.51, 0.32) | -0.02 (-0.45, 0.42) | 0.13 (-0.34, 0.60) | 0.03 (-0.07, 0.12) | 0.46 |
| Multivariable-adjusted animal protein substitution^6^ | 0.0 (Ref) | -0.22  (-0.62, 0.17) | -0.18 (-0.58, 0.21) | -0.18 (-0.61, 0.25) | -0.05 (-0.13, 0.04) | 0.48 |
| Percent Energy from Animal Protein | | | | | | |
| Mean % energy | 0.0 | 1.2 | 2.9 | 5.7 | 2.4 |  |
| Age- and sex-adjusted | 0.0 (Ref) | 0.10 (-0.28, 0.47) | 0.17 (-0.21, 0.54) | 0.53* (0.16, 0.91) | 0.09* (0.04, 0.15) | < 0.01 |
| Multivariable-adjusted | 0.0 (Ref) | -0.03  (-0.41, 0.35) | -0.06  (-0.44, 0.33) | 0.17  (-0.23, 0.57) | 0.04  (-0.02, 0.10) | 0.40 |
| Multivariable-adjusted carbohydrate substitution | 0.0 (Ref) | -0.00 (-0.40, 0.39) | -0.01 (-0.45, 0.43) | 0.25 (-0.31, 0.80) | 0.08 (-0.01, 0.17) | 0.40 |
| Multivariable-adjusted plant protein substitution^7^ | 0.0 (Ref) | -0.02 (-0.42, 0.37) | -0.06 (-0.49, 0.37) | 0.13 (-0.39, 0.64) | 0.05 (-0.04, 0.13) | 0.63 |

* P<.05

** P < .001

1. 501 overweight and obese particpants were excluded from this analysis. Underweight is defined as BMI < 18.5. Normal weight is defined as 18.5 ≤ BMI < 25. Overweight/Obese is defined as BMI ≥ 25.

2. Beta coefficients are in kg/m^2^ per 1% difference in percent energy

3. The age- and sex-adjusted model is adjusted for female's age (years, continuous) and sex (female, male)

4. The multivariable-adjusted model is adjusted for female's age, sex, female’s educational attainment (never attended school, completed primary or less, completed higher than primary) , if female is married or living with partner (yes, no), wealth index (poorest, poor, wealthy, wealthiest), female’s religion (orthodox Christian or not orthodox Christian), household size (continuous) and female employed (yes, no).

5. The multivariable-adjusted carbohydrate substitution model is the multivariable-adjusted model additionally adjusted for percentage of calories from total fat (continuous). When plant protein is the exposure, the model is additionally adjusted for percentage of calories from animal protein. When animal protein is the exposure, the model is additionally adjusted for percentage of calories from plant protein.

6. The multivariable-adjusted animal protein substitution model is adjusted for female's age, sex, percentage of calories from fat, percentage of calories from carbohydrate (continuous), female’s educational attainment, if female is married or living with partner, wealth index, female’s religion, household size and female employed.

7. The multivariable-adjusted plant protein substitution model is adjusted for the same covariates as the multivariable-adjusted animal protein substitution model.

**Supplementary Table 4**. Beta coefficients (95% confidence intervals) for percent energy intake from total, plant and animal protein in relation to body mass index (BMI) among 1,476 normal weight, overweight and obese adults^1^ in Addis Ababa, Ethiopia

|  | Q1 | Q2 | Q3 | Q4 | Per 1% difference^2^ | P_trend_ |
| --- | --- | --- | --- | --- | --- | --- |
| Percent Energy from Total Protein | | | | | | |
| Mean % energy | 11.8 | 13.6 | 14.8 | 16.5 | 14.2 |  |
| Age- and sex-adjusted^3^ | 0.0 (Ref) | 0.27  (-0.25, 0.80) | -0.16  (-0.69, 0.36) | 0.65*  (0.12, 1.18) | 0.08  (-0.02, 0.18) | 0.07 |
| Multivariable-adjusted^4^ | 0.0 (Ref) | 0.12  (-0.40, 0.64) | -0.39  (-0.91, 0.13) | 0.30  (-0.23, 0.83) | -0.00  (-0.10, 0.10) | 0.62 |
| Multivariable-adjusted carbohydrate substitution^5^ | 0.0 (Ref) | -0.00  (-0.53, 0.53) | -0.55*  (-1.08, -0.01) | 0.02  (-0.56, 0.60) | -0.07  (-0.19, 0.04) | 0.60 |
| Percent Energy from Plant Protein | | | | | | |
| Mean % energy | 9.4 | 11.1 | 12.3 | 14.1 | 11.7 |  |
| Age- and sex-adjusted | 0.0 (Ref) | -0.48 (-0.99, 0.04) | -0.22 (-0.74, 0.30) | -0.63* (-1.15, -0.11) | -0.13* (-0.23, -0.03) | 0.04 |
| Multivariable-adjusted | 0.0 (Ref) | -0.30 (-0.82, 0.22) | 0.11 (-0.42, 0.63) | -0.04 (-0.58, 0.50) | -0.01 (-0.11, 0.10) | 0.82 |
| Multivariable-adjusted carbohydrate substitution | 0.0 (Ref) | -0.28 (-0.83, 0.27) | -0.05 (-0.53, 0.64) | -0.11 (-0.75, 0.54) | -0.03 (-0.16, 0.11) | 0.99 |
| Multivariable-adjusted animal protein substitution^6^ | 0.0 (Ref) | -0.15 (-0.68, 0.38) | 0.24 (-0.30, 0.78) | 0.14 (-0.44, 0.73) | 0.04 (-0.08, 0.16) | 0.39 |
| Percent Energy from Animal Protein | | | | | | |
| Mean % energy | 0.0 | 1.2 | 2.9 | 5.8 | 2.5 |  |
| Age- and sex-adjusted | 0.0 (Ref) | 0.83* (0.31, 1.36) | 0.62* (0.10, 1.15) | 0.91** (0.39, 1.43) | 0.12* (0.04, 0.20) | < 0.01 |
| Multivariable-adjusted | 0.0 (Ref) | 0.51  (-0.02, 1.04) | 0.08  (-0.46, 0.63) | 0.13  (-0.43, 0.69) | 0.00  (-0.08, 0.09) | 0.83 |
| Multivariable-adjusted carbohydrate substitution | 0.0 (Ref) | 0.31 (-0.24, 0.85) | -0.30 (-0.90, 0.31) | -0.52 (-1.28, 0.23) | -0.11 (-0.24, 0.02) | 0.06 |
| Multivariable-adjusted plant protein substitution^7^ | 0.0 (Ref) | 0.30 (-0.25, 0.84) | -0.31 (-0.91, 0.29) | -0.54 (-1.25, 0.17) | -0.11  (-0.23, 0.00) | 0.04 |

* P<.05

** P < .001

1. 148 underweight particpants were excluded from this analysis. Underweight is defined as BMI < 18.5. Normal weight is defined as 18.5 ≤ BMI < 25. Overweight/Obese is defined as BMI ≥ 25.

2. Beta coefficients are in kg/m^2^ per 1% difference in percent energy

3. The age- and sex-adjusted model is adjusted for female's age (years, continuous) and sex (female, male)

4. The multivariable-adjusted model is adjusted for female's age, sex, female’s educational attainment (never attended school, completed primary or less, completed higher than primary) , if female is married or living with partner (yes, no), wealth index (poorest, poor, wealthy, wealthiest), female’s religion (orthodox Christian or not orthodox Christian), household size (continuous) and female employed (yes, no).

5. The multivariable-adjusted carbohydrate substitution model is the multivariable-adjusted model additionally adjusted for percentage of calories from total fat (continuous). When plant protein is the exposure, the model is additionally adjusted for percentage of calories from animal protein. When animal protein is the exposure, the model is additionally adjusted for percentage of calories from plant protein.

6. The multivariable-adjusted animal protein substitution model is adjusted for female's age, sex, percentage of calories from fat, percentage of calories from carbohydrate (continuous), female’s educational attainment, if female is married or living with partner, wealth index, female’s religion, household size and female employed.

7. The multivariable-adjusted plant protein substitution model is adjusted for the same covariates as the multivariable-adjusted animal protein substitution model.

**Supplementary Table 5.** Odds Ratios (95% confidence intervals) for associations of total energy intake with prevalence of overweight/obesity and underweight; and for associations of percent energy intake from total, plant and animal protein in relation to prevalence of overweight/obesity and underweight adjusted for total energy intake among 1,624 adults in Addis Ababa, Ethiopia

|  | Total Energy Intake | | | |  |  |
| --- | --- | --- | --- | --- | --- | --- |
|  | Q1 | Q2 | Q3 | Q4 | Per 100 kcal increment | P_trend_ |
| **Overweight/Obese**^1^  ^N=1476, 501 overweight or obese^ |  |  |  |  |  |  |
| Age and sex adjusted^2^ | 1.00 (Ref) | 0.98  (0.72, 1.35) | 1.04  (0.76, 1.43) | 1.07  (0.78, 1.46) | 1.00  (0.98, 1.02) | 0.60 |
| Multivariable-adjusted^3^ | 1.00 (Ref) | 0.83  (0.60, 1.15) | 0.85  (0.61, 1.17) | 0.83  (0.60, 1.15) | 0.98  (0.96, 1.00) | 0.33 |
| **Underweight^1^**  ^N=1123, 148 underweight^ |  |  |  |  |  |  |
| Age and sex adjusted | 1.00 (Ref) | 0.82  (0.51, 1.33) | 0.88  (0.54, 1.43) | 0.92  (0.56, 1.51) | 1.00  (0.97, 1.03) | 0.83 |
| Multivariable-adjusted | 1.00 (Ref) | 0.85  (0.52, 1.39) | 0.96  (0.59, 1.58) | 1.05  (0.63, 1.75) | 1.01  (0.98, 1.04) | 0.77 |
|  | Percent Energy from Total Protein | | | |  |  |
|  | Q1 | Q2 | Q3 | Q4 | Per 1% difference | P_trend_ |
| **Overweight/Obese**  ^N=1476, 501 overweight or obese^ |  |  |  |  |  |  |
| Age, sex and energy-adjusted^4^ | 1.00 (Ref) | 1.26 (0.91, 1.74) | 0.88  (0.63, 1.23) | 1.27  (0.91, 1.78) | 1.01  (0.94, 1.07) | 0.41 |
| Multivariable energy-adjusted^5^ | 1.00 (Ref) | 1.19  (0.86, 1.67) | 0.80  (0.57, 1.13) | 1.09  (0.77, 1.55) | 0.97  (0.91, 1.04) | 0.93 |
| Multivariable energy-adjusted carbohydrate substitution^6^ | 1.00 (Ref) | 1.14  (0.81, 1.59) | 0.74  (0.52, 1.06) | 0.95  (0.66, 1.38) | 0.93  (0.87, 1.00) | 0.39 |
| **Underweight**  ^N=1123, 148 underweight^ |  |  |  |  |  |  |
| Age, sex and energy-adjusted | 1.00 (Ref) | 0.77  (0.47, 1.26) | 0.57*  (0.34, 0.96) | 0.94  (0.57, 1.55) | 0.96  (0.86, 1.06) | 0.53 |
| Multivariable energy-adjusted | 1.00 (Ref) | 0.80  (0.48, 1.32) | 0.58*  (0.34, 0.99) | 0.96  (0.56, 1.59) | 0.96  (0.86, 1.07) | 0.57 |
| Multivariable energy-adjusted carbohydrate substitution | 1.00 (Ref) | 0.79  (0.48, 1.31) | 0.58*  (0.34, 0.99) | 0.94  (0.54, 1.61) | 0.95  (0.84, 1.06) | 0.49 |
|  | Percent Energy from Plant Protein | | | |  |  |
| **Overweight/Obese**  ^N=1476, 501 overweight or obese^ |  |  |  |  |  |  |
| Age, sex and energy-adjusted | 1.00 (Ref) | 0.76  (0.56, 1.04) | 0.88  (0.64, 1.20) | 0.78  (0.57, 1.07) | 0.94  (0.89, 1.00) | 0.20 |
| Multivariable energy-adjusted | 1.00 (Ref) | 0.82  (0.60, 1.13) | 1.10  (0.79, 1.52) | 1.12  (0.80, 1.56) | 1.02  (0.95, 1.09) | 0.32 |
| Multivariable energy-adjusted carbohydrate substitution | 1.00 (Ref) | 0.79  (0.56, 1.12) | 1.00  (0.69, 1.46) | 0.98  (0.65, 1.48) | 0.98  (0.90, 1.07) | 0.77 |
| Multivariable energy-adjusted animal protein substitution^7^ | 1.00 (Ref) | 0.92  (0.66, 1.28) | 1.26  (0.90, 1.77) | 1.39  (0.96, 2.00) | 1.07  (0.99, 1.15) | 0.04 |
| **Underweight**  ^N=1123, 148 underweight^ |  |  |  |  |  |  |
| Age, sex and energy-adjusted | 1.00 (Ref) | 1.00  (0.59, 1.69) | 1.51  (0.92, 2.49) | 1.29  (0.77, 2.14) | 1.08  (0.98, 1.19) | 0.19 |
| Multivariable energy-adjusted | 1.00 (Ref) | 0.97  (0.57, 1.66) | 1.36  (0.81, 2.27) | 1.08  (0.63, 1.83) | 1.04  (0.94, 1.15) | 0.61 |
| Multivariable energy-adjusted carbohydrate substitution | 1.00 (Ref) | 0.88  (0.49, 1.57) | 1.15  (0.63, 2.08) | 0.87  (0.46, 1.66) | 0.99  (0.87, 1.13) | 0.77 |
| Multivariable energy-adjusted animal protein substitution | 1.00 (Ref) | 1.03  (0.59, 1.82) | 1.45  (0.85, 2.50) | 1.24  (0.69, 2.25) | 1.08  (0.96, 1.21) | 0.33 |
| Percent Energy from Animal Protein | | | | | |  |
| **Overweight/Obese**  ^N=1476, 501 overweight or obese^ |  |  |  |  |  |  |
| Age, sex and energy-adjusted | 1.00 (Ref) | 1.46*  (1.05, 2.03) | 1.39  (0.99, 1.95) | 1.36  (0.97, 1.95) | 1.04  (0.99, 1.09) | 0.23 |
| Multivariable energy-adjusted | 1.00 (Ref) | 1.21  (0.85, 1.70) | 1.01  (0.70, 1.45) | 0.85  (0.59, 1.29) | 0.97  (0.92, 1.03) | 0.13 |
| Multivariable energy-adjusted carbohydrate substitution | 1.00 (Ref) | 1.08  (0.76, 1.54) | 0.80  (0.54, 1.20) | 0.56*  (0.34, 0.91) | 0.90*  (0.83, 0.98) | < 0.01 |
| Multivariable energy-adjusted plant protein substitution^7^ | 1.00 (Ref) | 1.09  (0.76, 1.55) | 0.82  (0.56, 1.22) | 0.59*  (0.37, 0.93) | 0.91*  (0.85, 0.98) | < 0.01 |
| **Underweight**  ^N=1123, 148 underweight^ |  |  |  |  |  |  |
| Age, sex and energy-adjusted | 1.00 (Ref) | 0.88  (0.54, 1.43) | 0.82  (0.49, 1.36) | 0.67  (0.40, 1.14) | 0.92*  (0.84, 1.00) | 0.14 |
| Multivariable energy-adjusted | 1.00 (Ref) | 0.91  (0.55, 1.51) | 0.91  (0.53, 1.54) | 0.81  (0.46, 1.42) | 0.94  (0.86, 1.03) | 0.48 |
| Multivariable energy-adjusted carbohydrate substitution | 1.00 (Ref) | 0.89  (0.53, 1.50) | 0.87  (0.48, 1.57) | 0.76  (0.36, 1.62) | 0.90  (0.79, 1.03) | 0.50 |
| Multivariable energy-adjusted plant protein substitution | 1.00 (Ref) | 0.90  (0.53, 1.50) | 0.87  (0.49, 1.54) | 0.75  (0.38, 1.51) | 0.91  (0.81, 1.03) | 0.44 |

*P<.05

1. Overweight/Obese is defined as BMI ≥ 25 and is compared with a reference category of non-overweight/obese (BMI < 25). All underweight participants (n=148) were excluded from the overweight analysis. Underweight is defined as BMI < 18.5 and is compared with a reference category of non-underweight (BMI ≥ 18.5). All overweight participants (n=501) were excluded from the underweight analysis.

2. The age- and sex-adjusted model is adjusted for female's age (years, continuous) and sex (female, male)

3. The multivariable-adjusted model is adjusted for female's age, sex, female’s educational attainment (never attended school, completed primary or less, completed higher than primary) , if female is married or living with partner (yes, no), wealth index (poorest, poor, wealthy, wealthiest), female’s religion (orthodox Christian or not orthodox Christian), household size (continuous) and female employed (yes, no).

4. The age, sex and energy-adjusted model is adjusted for female's age (years, continuous), sex (female, male) and total energy intake (kcal, continous)

5. The multivariable energy-adjusted model is the multivariable-adjusted model additionally adjusted for total energy intake

6. The multivariable energy-adjusted carbohydrate substitution model is the multivariable energy-adjusted model additionally adjusted for percentage of calories from total fat (continuous). When plant protein is the exposure, the model is additionally adjusted for percentage of calories from animal protein. When animal protein is the exposure, the model is additionally adjusted for percentage of calories from plant protein.

7. The multivariable energy-adjusted animal protein substitution model and the multivariable energy-adjusted plant protein substitution models are both adjusted for total energy intake, female's age, sex, percentage of calories from fat, percentage of calories from carbohydrate (continuous), female’s educational attainment, if female is married or living with partner, wealth index, female’s religion, household size and female employed.

**Supplementary Table 6.** Difference between coefficients and 95% confidence intervals for percent of total energy intake (%TEI) from macronutrients from the energy partition model for outcomes of overweight/obesity^1^, underweight^2^ and continuous body mass index (BMI)

| Comparison | | Difference between coefficients (95% CI) | P value |
| --- | --- | --- | --- |
| Overweight/obesity (excluding underweight participants), n=1,476 | | | |
| Crude | %TEI protein and %TEI carb | -0.06 (-0.12, 0.01) | 0.11 |
|  | %TEI fat and %TEI carb | 0.05 (0.02, 0.07) | <.001 |
|  | %TEI protein and %TEI fat | -0.10 (-0.18, -0.02) | 0.01 |
| Multivariable-adjusted^3^ | %TEI protein and %TEI carb | -0.08 (-0.16, -0.01) | 0.02 |
|  | %TEI fat and %TEI carb | 0.03 (0.00, 0.05) | 0.02 |
|  | %TEI protein and %TEI fat | -0.11 (-0.20, -0.03) | 0.01 |
| Underweight (excluding overweight/obese participants), n=1,123 | | | |
| Crude | %TEI protein and %TEI carb | -0.07 (-0.18, 0.04) | 0.21 |
|  | %TEI fat and %TEI carb | 0.00 (-0.03, 0.04) | 0.80 |
|  | %TEI protein and %TEI fat | -0.07 (-0.21, 0.57) | 0.27 |
| Multivariable-adjusted | %TEI protein and %TEI carb | -0.05 (-0.16, 0.06) | 0.39 |
|  | %TEI fat and %TEI carb | 0.15 (-0.02, 0.05) | 0.44 |
|  | %TEI protein and %TEI fat | -0.06 (-0.20, -0.07) | 0.35 |
| Continous BMI (full sample), n=1,624 | | | |
| Crude | %TEI protein and %TEI carb | -0.01 (-0.13, 0.11) | 0.88 |
|  | %TEI fat and %TEI carb | 0.09 (0.05, 0.13) | <.001 |
|  | %TEI protein and %TEI fat | -0.10 (-0.25, 0.05) | 0.18 |
| Multivariable-adjusted | %TEI protein and %TEI carb | -0.06 (-0.18, 0.06) | 0.30 |
|  | %TEI fat and %TEI carb | 0.05 (0.01, 0.09) | 0.01 |
|  | %TEI protein and %TEI fat | -0.11 (-0.26, 0.03) | 0.12 |

1. Overweight/Obese is defined as BMI ≥ 25.

2. Underweight is defined as BMI < 18.5.

3. The multivariable-adjusted model is adjusted for female's age, sex, female’s educational attainment (never attended school, completed primary or less, completed higher than primary) , if female is married or living with partner (yes, no), wealth index (poorest, poor, wealthy, wealthiest), female’s religion (orthodox Christian or not orthodox Christian), household size (continuous) and female employed (yes, no).
