# Supplementary figures and images for: Associations of percentage energy intake from total, animal and plant protein with overweight/obesity and underweight among adults in Addis Ababa, Ethiopia
Source: Public Health Nutr. 2022 May 16;25(11):3107–20. doi: 10.1017/S1368980022001100 (PMC9991810; doi:10.1017/S1368980022001100)

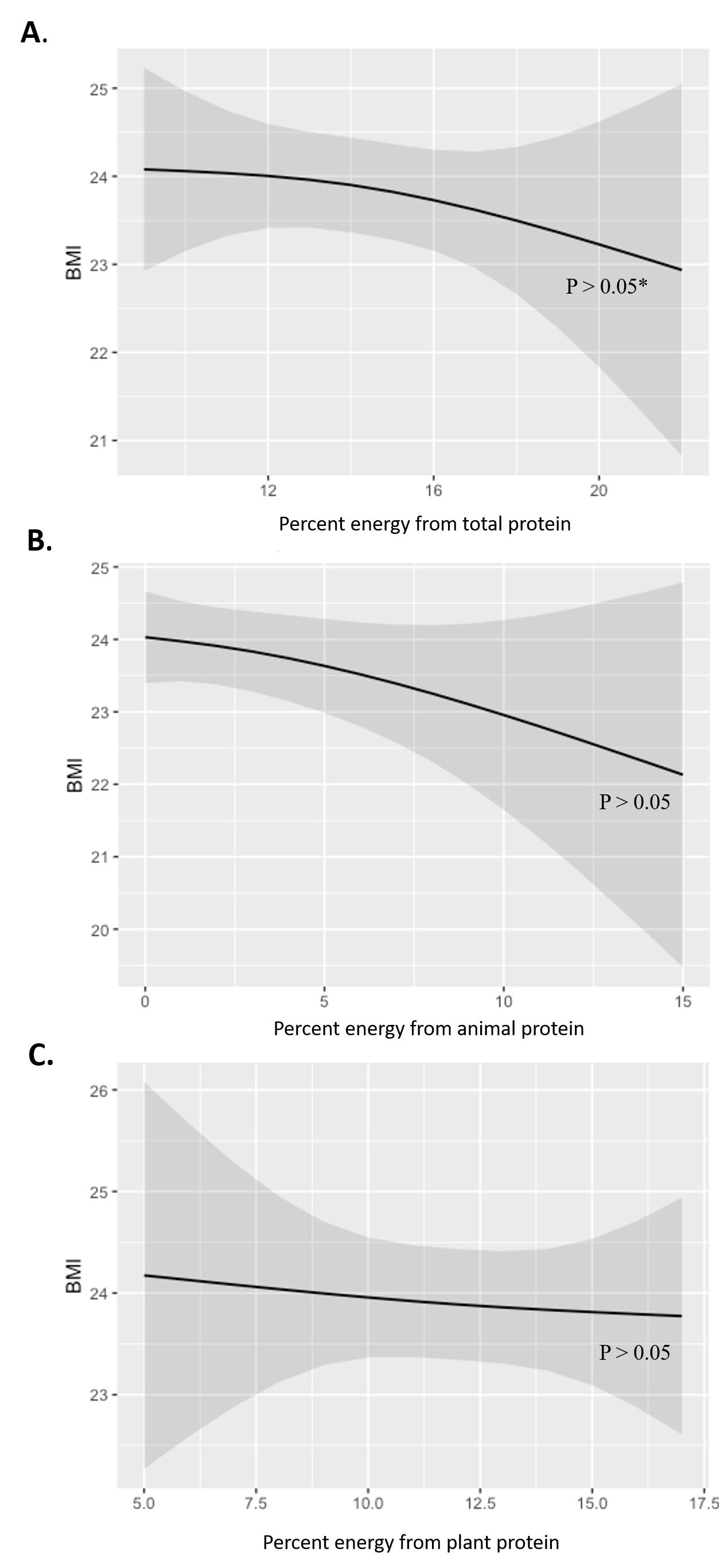

Supplement: Supplementary file 1 [file S1368980022001100sup.zip › S1368980022001100sup001.tif]

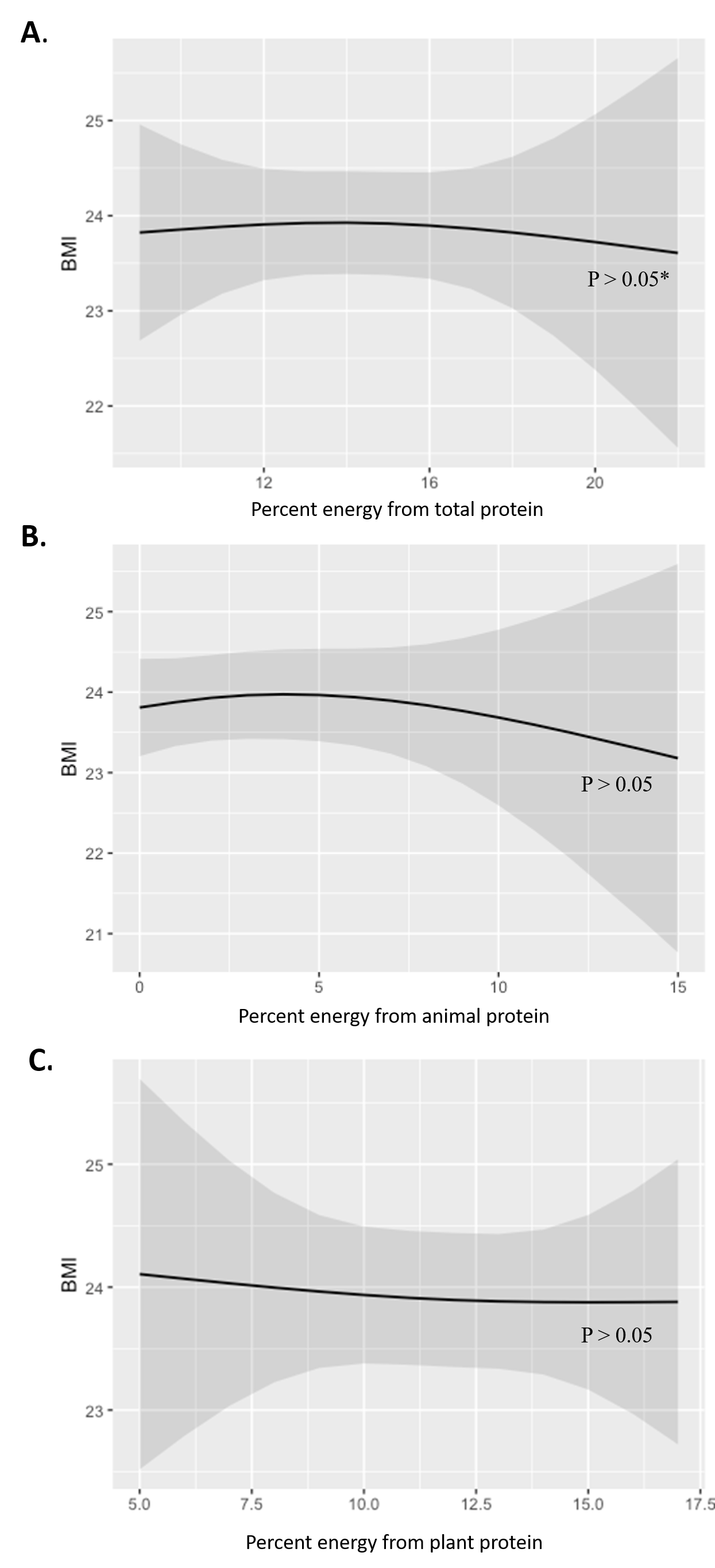

Supplement: Supplementary file 1 [file S1368980022001100sup.zip › S1368980022001100sup002.tif]
